# Neural innervation as a potential trigger of morphological color change and sexual dimorphism in cichlid fish

**DOI:** 10.1038/s41598-020-69239-w

**Published:** 2020-07-23

**Authors:** Yipeng Liang, Axel Meyer, Claudius F. Kratochwil

**Affiliations:** Zoology and Evolutionary Biology, Department of Biology, University of Konstanz, Universitätsstrasse 10, 78457 Konstanz, Germany

**Keywords:** Evolutionary developmental biology, Ichthyology

## Abstract

Many species change their coloration during ontogeny or even as adults. Color change hereby often serves as sexual or status signal. The cellular and subcellular changes that drive color change and how they are orchestrated have been barely understood, but a deeper knowledge of the underlying processes is important to our understanding of how such plastic changes develop and evolve. Here we studied the color change of the Malawi golden cichlid (*Melanchromis auratus*). Females and subordinate males of this species are yellow and white with two prominent black stripes (yellow morph; female and non-breeding male coloration), while dominant males change their color and completely invert this pattern with the yellow and white regions becoming black, and the black stripes becoming white to iridescent blue (dark morph; male breeding coloration). A comparison of the two morphs reveals that substantial changes across multiple levels of biological organization underlie this polyphenism. These include changes in pigment cell (chromatophore) number, intracellular dispersal of pigments, and tilting of reflective platelets (iridosomes) within iridophores. At the transcriptional level, we find differences in pigmentation gene expression between these two color morphs but, surprisingly, 80% of the genes overexpressed in the dark morph relate to neuronal processes including synapse formation. Nerve fiber staining confirms that scales of the dark morph are indeed innervated by 1.3 to 2 times more axonal fibers. Our results might suggest an instructive role of nervous innervation orchestrating the complex cellular and ultrastructural changes that drive the morphological color change of this cichlid species.

## Introduction

Coloration is an important feature that plays crucial roles in terms of natural and sexual selection. It can serve in predator avoidance, prey capture through camouflage, conspecific communication and protection from radiation^1,2^. Besides these ecological and evolutionary aspects, the formation of pigment patterns provides insights into the genetic basis of adaptive evolution^3^ as well as the formation of complex tissues^4^. In vertebrates the color of the integument is shaped by the multilayered arrangement and interaction of cells with different pigmentary and structural properties^5^. Variations in the density, shape and properties of chromatophores and the intracellular organization of pigments and reflective molecules shapes the macroscopic appearance of the integument^6,7^. Several types of pigment-bearing and light-reflecting chromatophores have been identified in teleosts^8^. The most common cell types are melanophores (containing the brown to black pigment melanin), xanthophores/erythrophores (containing yellow to red pigments including carotenoids and pteridines), leucophores (white) and iridophores (containing guanine platelet crystals that produce structural coloration)^9^.

Although coloration is often perceived and studied as a static trait, it can change very dynamically on different time scales during the lifetime of an organism. Rapid changes (milliseconds to hours) are referred to as physiological color change and are triggered by neuronal and hormonal signals. These signals cause changes in the intracellular distribution of pigments or reflective molecules. In contrast, changes that involve differences in pigment quantity or cell number are referred to as morphological color change and occur over longer periods of time (hours to months)^8,10,11^.

**Figure 1:**
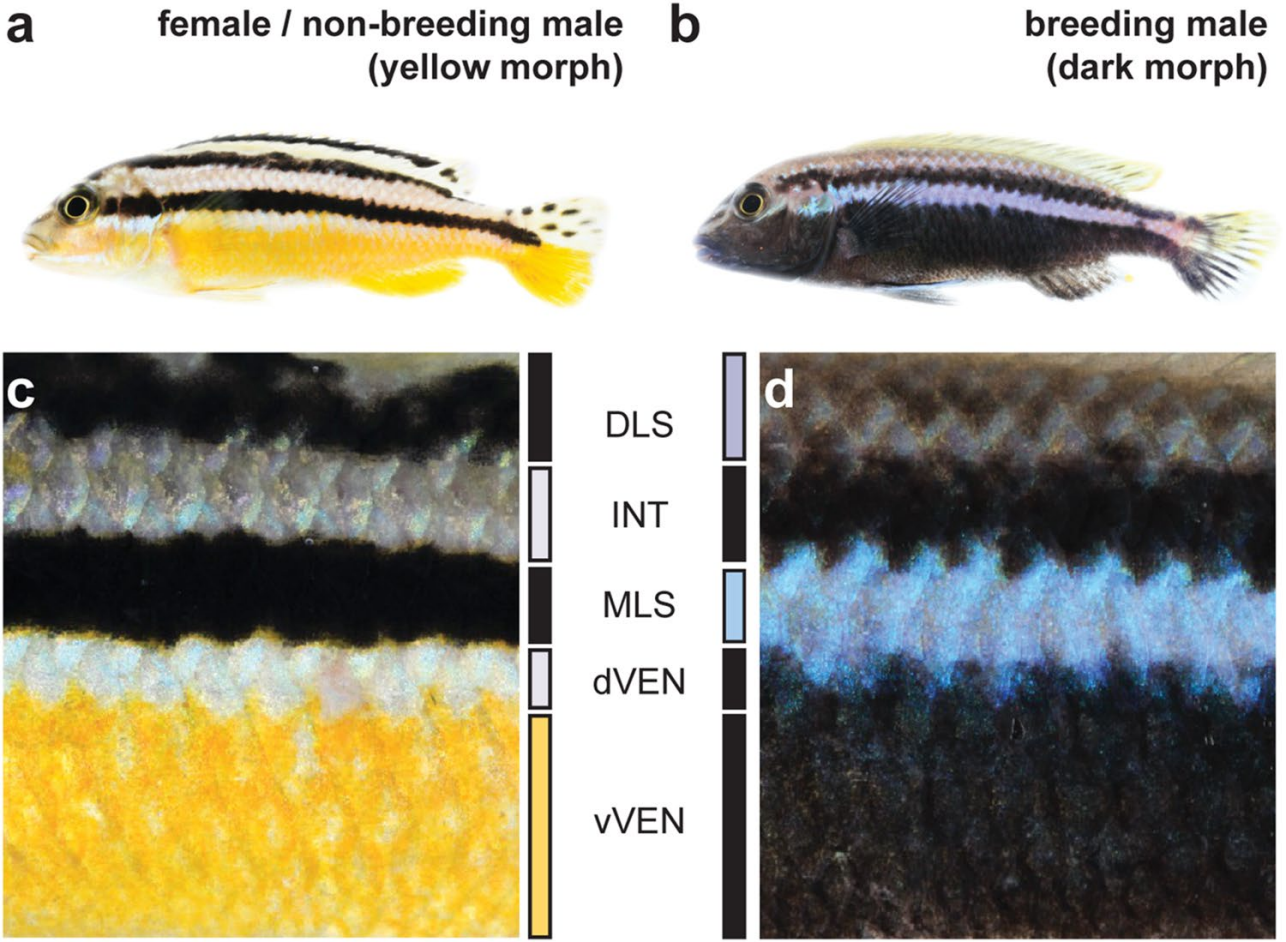
Yellow and dark morph of *Melanochromis auratus*. (**a**,**b**) Females and subordinate males of *M. auratus* (**a**) are brightly yellow colored with two black stripes (yellow morph). Dominant males (**b**) transform into the dark morph that has two grey to blue stripes on a black background (dark morph). (**c**,**d)** To comparatively analyze the skin of the yellow (**c**) and dark (**d**) morph we defined five homologous regions: The dorsolateral stripe (DLS, black in yellow morph, purple/blue in dark morph), the interstripe (INT, white/gray in yellow morph*,* black in dark morph), the midlateral stripe (MLS, black in yellow morph, blue in dark morph), the dorsal part of the ventral integument (dVEN, white in yellow morph, black in dark morph) and the ventral part of the ventral integument (vVEN, yellow in yellow morph, black in dark morph).

Teleost fishes lend themselves well for studying the developmental and cellular mechanisms and the molecular bases of color and color change phenotypes^12–15^. With their richness in pigmentation patterns, fishes in the family Cichlidae (cichlid fishes) are an attractive model for studying how genomic changes facilitate variation in molecular and developmental mechanisms and thereby the evolution of pigmentation phenotypes^15–20^. Cichlid fishes also offer remarkable examples for morphological color change, color polymorphism and polyphenisms as well as sexual dichromatism, traits that have received much less attention, especially from a molecular and cellular perspective. For example, in the Central American Midas cichlid *Amphilophus citrinellus*, 5 to 10% of the individuals lose their typical dark pigmentation and obtain a “golden” orange sometimes also white, yellow or red coloration^21,22^. In the haplochromine cichlid *Astatotilapia burtoni*, the coloration of males serves as a social signal and can change between bright blue and bright yellow^23,24^. This color change is regulated by the neuroendocrine melanocortin system: the yellowness of the fish body is hereby modulated by α-melanocyte-stimulating hormone (α-MSH)^25^. Another striking case of morphological color change occurs in the Malawi golden cichlid *Melanochromis auratus* (Fig. 1a,b)^26,27^ and was described to be the “perhaps most remarkable case” of “color change reported for any cichlid”^26^. *M. auratus* is a species endemic to the southern part of Lake Malawi. However, while the ecology^28^, phylogeny^29^, populations genetics^30^ and the sexual dimorphism^31^ of the species have been thoroughly investigated since it’s discovery in 1897 by George Albert Boulenger^32^, the color change and its underlying molecular mechanisms have not been studied, so far. While juveniles, females and subordinate males of *M. auratus* are bright yellow with two melanic horizontal stripes that is referred to as yellow morph^26^ (Fig. 1a,c), dominant males undergo a drastic morphological color change and become dark with two light blue horizontal stripes (dark morph; Fig. 1b,d).

With a few exceptions as for example the recent investigation of seasonal camouflage in snowshoe hares^33^, the molecular mechanisms and genetic control of color change remain barely understood^34,35^. A detailed understanding of such extreme examples, where we observe complex changes in adult characteristics, will give insights into how such changes can be orchestrated, how they manifest as well as what levels of biological organization are mechanistically involved. Moreover, they might also provide a unique opportunity understand the molecular mechanism that underly the evolution of phenotypic plasticity^36^ and sexual dimorphisms^37^.

To specifically test what levels of biological organization are involved in driving the color change of *M. auratus* we comprehensively analyze how ultrastructural (using transmission electron microscopy), cellular (using light microscopy and immunohistochemistry) and transcriptomic (using RNA-sequencing) changes contribute to these remarkable differences in adult morphology. Hereby, our work reveals a surprising association of morphological color change with increased neural innervation. Taken together, our results provide novel insights into the cellular, and molecular underpinnings of a remarkable case of morphological color change that differentiates both females and male subordinates from dominant males.

## Results

### Chromatophore number, organization and properties differ between yellow and dark morph of *M. auratus*

Both yellow morph and dark morph of *M. auratus* are characterized by two longitudinal (horizontal) stripes (Fig. 1a,b). As a first step, we histologically compared the two morphs and defined five regions across dorsal–ventral axis that differ in their coloration in the two morphs (Fig. 1c,d): dorsolateral stripe (DLS), interstripe (INT), midlateral stripe (MLS), the dorsal portion of the ventral region (dVEN), and the ventral portion of the ventral region (vVEN).

**Figure 2:**
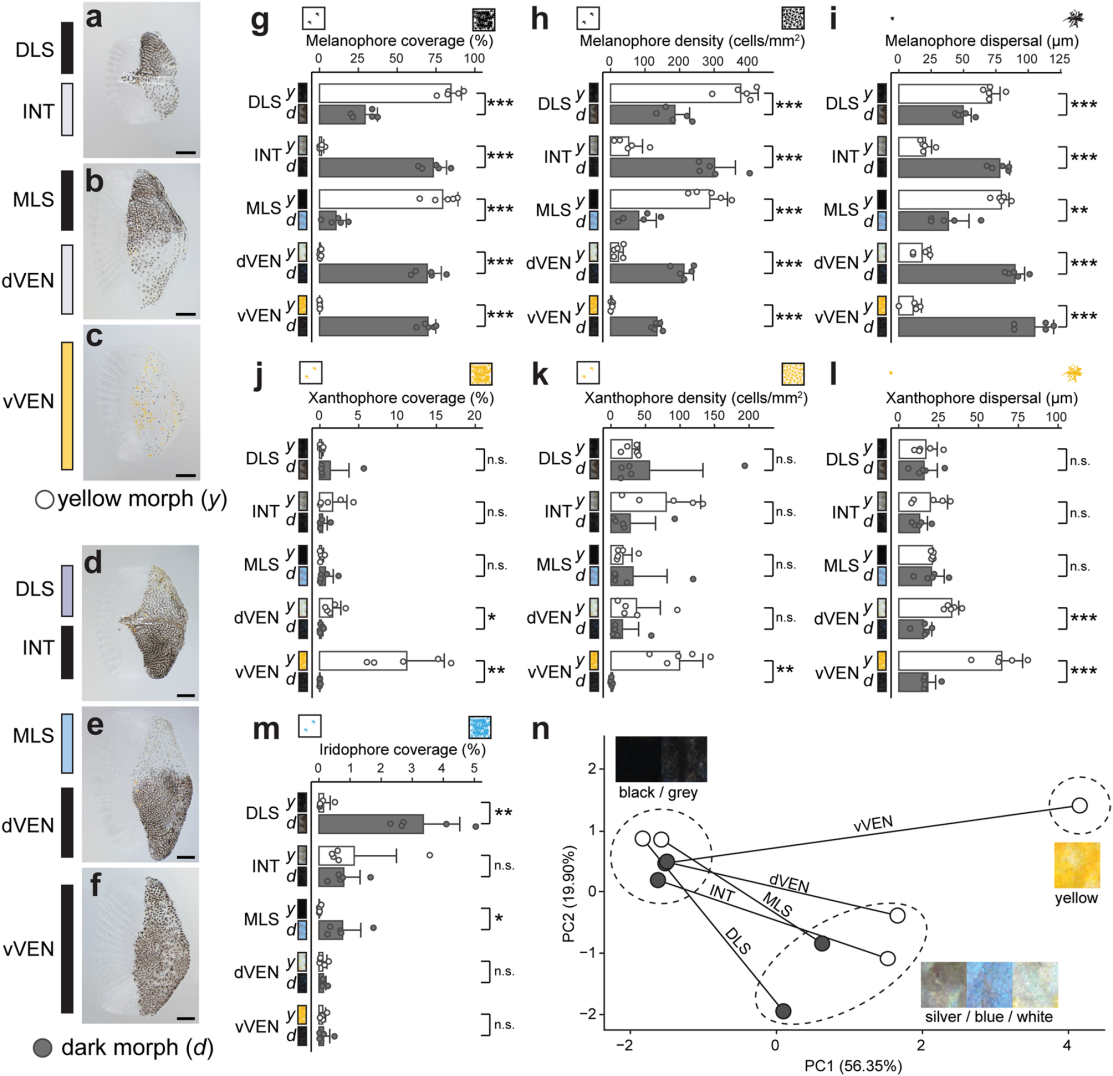
Chromatophore measurements in scales of the yellow and dark morph of *M. auratus*. (**a**–**f)** Photographs of individual scales from yellow morph (**a**–**c**) and dark morph (**d**–**f**). Scale bars correspond to 500 μm. (**g**–**i**) Measurements for melanophores in the five homologous regions (DLS, INT, MLS, dVEN and vVEN) of yellow and dark morph including coverage (**g**), cell density (**h**) and dispersal (**i**). (**j**–**l**) Same measurements for xanthophores including coverage (**j**), cell density (**k**) and dispersal (**l**). (**m**) Measurement of iridophore coverage. Differences between morphs in the same homologous regions were evaluated by two-tailed *t* test, n = 5 (individual points). Each point represents one individual (mean value of five scales). Error bars indicate means + SD. Significant sign: *** *P* < 0.001, **P < 0.01, *P < 0.05, n.s. non-significant. Full data for (**g**–**m**) see Supplementary Tables S1, S2. (**n**) Principal component analysis (PCA) using all measurements, demonstrating that similarly colored regions cluster together and that the morphological changes of the stripe regions (MLS, DLS) and other regions (INT, dVEN, vVEN) between yellow (white circles) and dark morph (dark circles) occur in opposite directions. PC1 mainly correlates with melanophore properties (negatively) and xanthophore properties (positively), PC2 correlates negatively with differences in iridophore coverage.

To test whether and how the morphological color change in *M. auratus* can be explained by changes in chromatophore number, distribution and characteristics, we compared chromatophores in yellow and dark morph using light microscopy of whole-mount scale preparations. In line with previous descriptions for cichlids^7,38^, three types of chromatophores could be detected in both morphs: melanophores with black to dark brown pigmentation, xanthophores with yellow to orange pigmentation, and iridophores that produce iridescent/reflective colors (Supplementary Fig. S1). To describe chromatophore distributions and characteristics we measured (a) chromatophore coverage (melanophores, xanthophores and iridophores), (b) chromatophore density (melanophores and xanthophores), and (c) chromatophore size (melanophores and xanthophores) in the epidermal layer that covers the scales (Fig. 2).

Chromatophore coverage was calculated by measuring the percentage of the scale that is covered by dark pigment (melanophores), yellow to orange pigment (xanthophores—detected by autofluorescence) and iridescent substances (iridophores—detected using polarized light). Chromatophore coverage is influenced both by number and size of the cells (as well as intracellular dispersal/aggregation of pigments) that we also quantified individually. Size of the pigmented area of melanophores and xanthophores (that is influenced both by cell size and melanosome/xanthosome dispersal within the cell) was estimated by measuring the diameter of the minimally sized circle that encloses all pigmented parts. To obtain reliable cell number estimates we treated scales with adrenaline to aggregate the melanosomes, thereby easing both quantifications of xanthophore and melanophore number (Supplementary Fig. S1). As cell delimitations could be only visualized for xanthophores and melanophores, for iridophores solely the chromatophore coverage was measured.

The most pronounced differences (see a more detailed report in the supplementary text) were found for melanophores and are consistent with the strong phenotypic differences in pigmentation (Figs. 1 and 2a–f), melanophore coverage, melanophore cell density, and average melanosome dispersal diameter differed significantly between dark and yellow morph (all *P* < 0.01, two-tailed *t* test; Fig. 2g–i, Supplementary Tables S1, S2). Differences in xanthophore coverage, xanthophore cell density and xanthophore size/dispersal were restricted to the ventral regions (vVEN and dVEN) (Fig. 2j–l, Supplementary Tables S1, S2). Although we could identify iridophores by polarized light illumination (Supplementary Fig. S1), we were not able to demarcate individual cells. Therefore, we only measured iridophore coverage but not density and diameter of iridophores. Iridophore coverage increased significantly in the two regions with iridescent white/blue coloration in the dark morph (DLS and MLS) (Fig. 2m, Supplementary Tables S1, S2). When all data were analyzed by a principal component analysis, we observe that the five homologous regions of the two morphs largely cluster by color (Fig. 2n).

### Three-dimensional arrangement of chromatophores and their properties shape the phenotypic differences between morphs

Differences in coloration between yellow and dark morph might likely not only be driven by chromatophore differences in scales but also in the underlying skin. While scales can be easily investigated using light microscopy as there is only one cell layer of chromatophores, in the underlying integument such investigation is more challenging due to the three-dimensional arrangement of chromatophores^39,40^. To address this problem, we used transmission electron microscopy (TEM) to examine the multilayered arrangement of chromatophores as well as the ultrastructure of the pigment-bearing and structural organelles.

All chromatophores could be classified using TEM. Melanophores were identified by the presence of melanin-containing, black melanosomes (Supplementary Fig. S1)^41,42^. Xanthophores could be recognized by their round pigment organelles that contain carotenoid and/or pteridine and appeared grey (in contrast to the dark melanosomes) on the TEM images (Supplementary Fig. S1)^40–42^. Iridophores are characterized by stacks of white layers (Supplementary Fig. S1)^40–44^. These areas indicate the position of iridosomes (also referred to as reflecting platelets) that are composed of guanine crystals (guanine crystals are lost during the sample preparation and therefore appear as white areas on TEM images)^43,44^. Iridosomes reflect light and can therefore, if orientated horizontally, intensify the colors of chromatophores on top of the platelets (i.e. the yellow to orange color of xanthophores). Tilted iridosome arrangements causing light interference can result in structural coloration^8,43,44^. We therefore hypothesized that the main differences in dark and yellow coloration will be explained by differences in melanophore and xanthophore numbers, respectively—or at a subcellular level by differences in melanosomes and xanthosomes. The shiny appearance of yellow and white regions might be caused by horizontally (or fully randomly) oriented guanine-platelets, while the iridescent blue coloration of the MLS of the dark morph might be due to a tilted, parallelly oriented and lamellar-like organized iridosomes.

**Figure 3:**
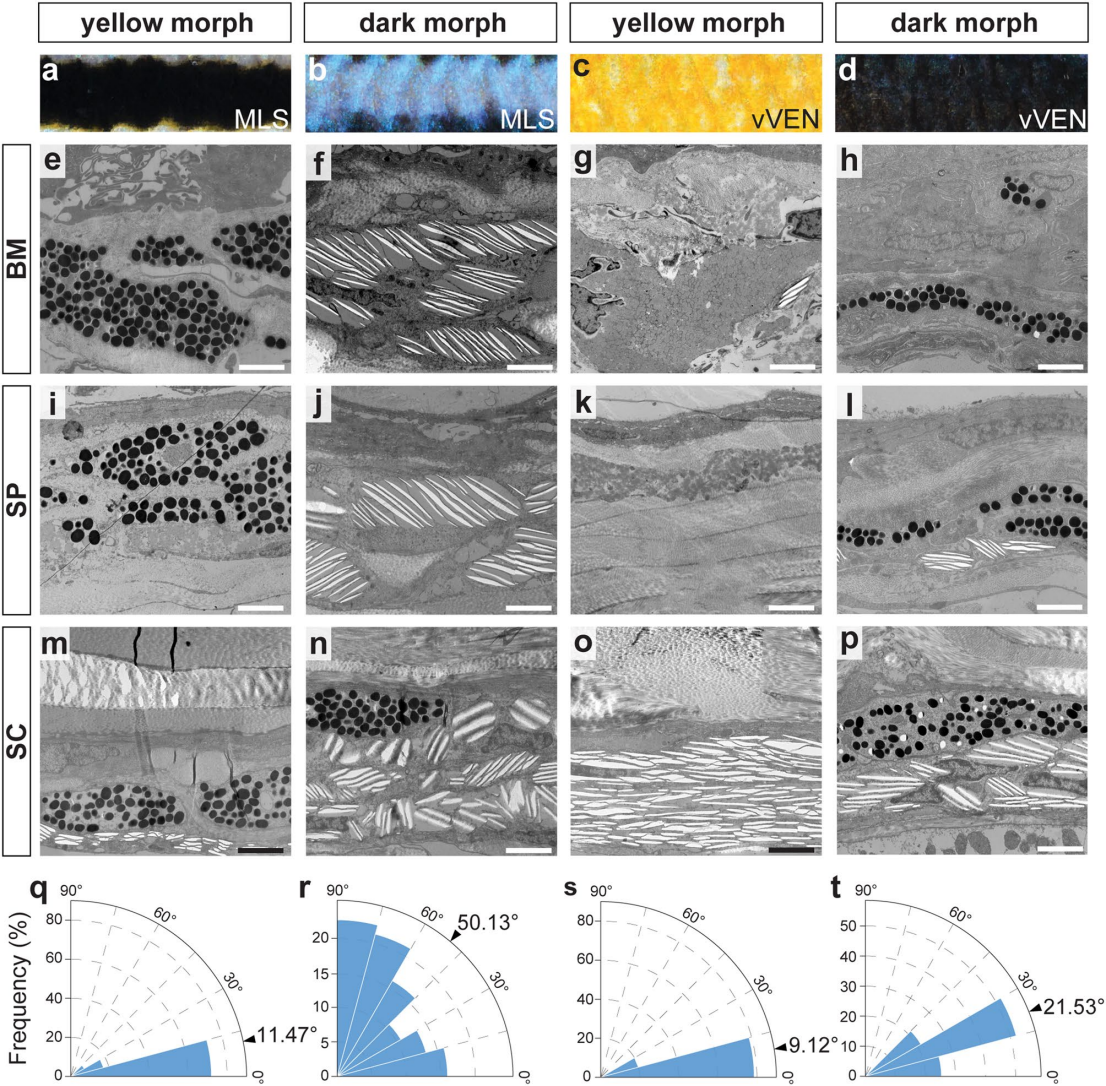
Ultrastructural differences between skin regions of the yellow and dark morph. (**a-d**) Macroscopic appearance of the two homologous regions MLS (**a**,**b**) and vVEN (**c**,**d**) that were comparatively analyzed between yellow (**a**,**c**) and dark (**b**,**d**) morph. (**e–p)** The ultrastructure of the integument was analyzed using transmission electron microscopy (TEM). Chromatophores were mainly found in three layers: Directly beneath the basal membrane (BM; **e**–**h**), within the loose connective tissue of the stratum spongiosum (SP; **i**–**l**) and in the deepest layer below the stratum compactum (SC; **m**–**p**). The layers were compared between the midlateral stripe (MLS) of the yellow (**e**,**i**,**m**) and dark morph (**f**,**j**,**n**) as well as the ventral part of the ventral integument (vVEN) of the yellow (**g**,**k**,**o**) and dark morph (**h**,**l**,**p**). Scale bars correspond to 2 μm. (**q**–**t)** Polar chart of the angle of iridosomes in the stratum compactum of the MLS of yellow (**q**) and dark morph (**r**) as well as the vVEN of the yellow (**s**) and dark morph (**t**). Quantifications of all layers in dark morph MLS can be found in Supplementary Fig. S3.

Chromatophores of *M. auratus* are organized in three layers in the integument (Fig. 3): (a) In the basal membrane layer (BM), the uppermost layer that is directly beneath the basal membrane (Fig. 3e–h), (b) in the stratum spongiosum layer (SP) that is the middle layer within the loose connective tissue of the upper dermis (Fig. 3i–l) and (c) in the stratum compactum layer (SC), the bottommost layer between the dense, collagenous layer of the stratum compactum and the vascular layer that delimits the dermis from the muscle (Fig. 3m–p).

Two main observations emerge: (1) Clear differences between the similarly dark-pigmented regions of both morphs [MLS of the yellow morph (Fig. 3a) and vVEN of the dark morph (Fig. 3d)] and (2) striking morphological differences between homologous, but differently colored regions [i.e. MLS, dark in the yellow morph (Fig. 3a) *vs.* iridescently blue in the dark morph (Fig. 3b); vVEN, brilliantly yellow in the yellow morph (Fig. 3c) *vs*. dark in the dark morph (Fig. 3d)].

### Similarly colored skin regions are caused by different cellular compositions

In dark regions of both morphs, e.g. MLS of the yellow morph (Fig. 3e,i,m) and vVEN of the dark morph (Fig. 3h,l,p), we found melanophores in all three chromatophore layers. The melanophores in dark-pigmented regions of both morphs were mostly in a dispersed state. Melanosome density is higher in MLS of the yellow morph (Supplementary Fig. S2) than in vVEN of the dark morph (Supplementary Fig. S2)—compatible with the stronger black appearance in MLS of the yellow morph (Fig. 3a). The melanophores in the bottommost layer of the yellow morph MLS and the dark morph vVEN were underlaid by iridophores with few iridosomes (Fig. 3q,t). Iridosomes are almost arranged parallel to the surface in the yellow morph MLS (mean angle to surface 11.47°) (Fig. 3q). In contrast, platelets are more tilted in the dark morph vVEN (mean angle to surface 21.53°) (Fig. 3t). In the two upper layers of the yellow morph MLS, melanophores were more scattered, thereby often overlaying small-sized xanthophores (Fig. 3e,i). In the SP of dark morph vVEN, small numbers of iridophores were found beneath the melanophores (Fig. 3l).

### Color changes of homologous regions are driven by structural and cellular modifications

For the vVEN of the yellow morph, which is brightly yellow colored, we found stacked and almost parallelly oriented reflecting platelets (mean angle to surface 9.12°) in the bottommost SC layer (Fig. 3o,s). The two upper layers, BM and SP, had numerous xanthophores (Fig. 3g,k). Melanophores were almost absent from all layers (Fig. 3g,k,o). The ventral area of the yellow morph therefore had more xanthophores, less melanophores and more and more parallel oriented iridosomes within iridophores in the SC. Usually iridosomes are quite thin (e.g. in the neon tetra, *Paracheirodon innesi*, that has been intensively studied in this respect, thickness ranges between 5 and 60 nm^45,46^). In *M. auratus* iridosomes were substantially thicker in all regions and both morphs (mean thickness between 94 and 144 nm; distance between iridosomes between 76 and 147 nm; Supplementary Fig. S4). This is in accordance with the hypothesis that such structural characteristics—that merely lead to a reflection of light in a mirror-like fashion—would result in the bright yellow coloration of the vVEN region of the yellow morph.

The most noticeable characteristic in the integument of the distinct bluish iridescent dark morph MLS (Fig. 1b) is that it is composed of several sheets of highly organized iridophores (Fig. 3f,j,n). In the upper layers, guanine platelets are arranged in parallel but strongly angled (mean angle to surface in BM: 26.10°; mean angle to surface in SP: 38.63°; Fig. 3f,I; Supplementary Fig. S3). In the bottom layer (SC) the orientation appears more random and essentially ranges from 0 to 90° (mean angle to surface: 50.13°; Fig. 3n,r). Some aggregated melanophores (Fig. 3n) were also found in the in the iridophore layers, especially in the SC layer. The highly organized ultrastructure of the iridophores in the dark morph MLS confirms the hypothesis that the iridescent blue coloration is caused by iridosome orientation in the integument.

Within the other dorso-ventral regions we found comparable patterns that are comprehensively summarized in Supplementary Fig. S5. The differences between the INT region of the yellow and dark morph resembled those of the vVEN region with the main difference being the much higher number of xanthophores in the vVEN of the yellow morph compared to the INT region. This is compatible with the whitish appearance of the INT region (Supplementary Fig. S5). The DLS region resembles the MLS region, with the main difference being that the DLS of the dark morph has less organized iridosomes likely resulting in the more greyish to whitish appearance of the DLS compared to the bluish iridescent MLS (Supplementary Fig. S5).

### Comparative transcriptomics of *M. auratus* color morphs

**Figure 4:**
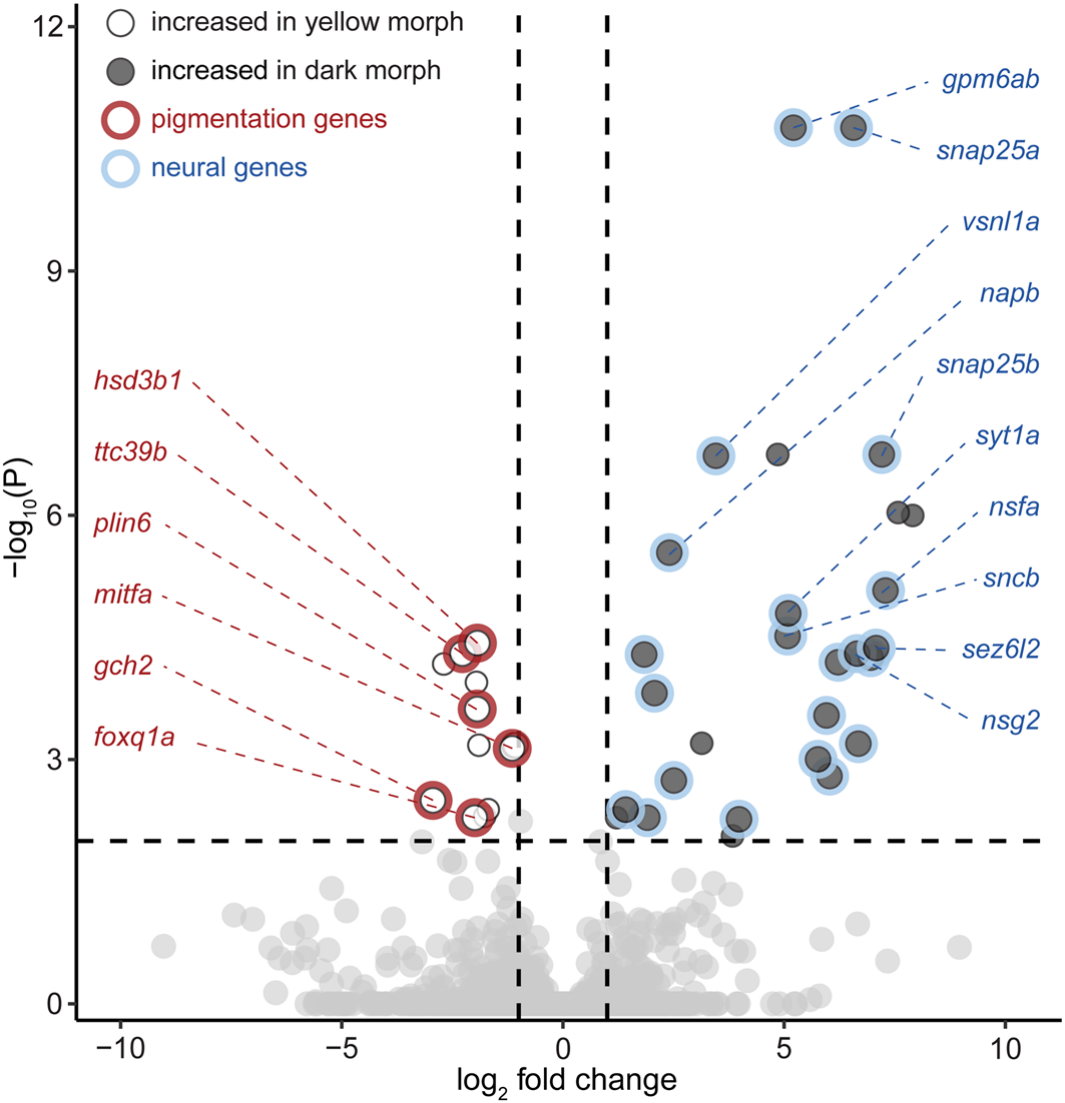
Differential gene expression analysis. Differential expression between yellow morph (n = 4) versus dark morph skin (n = 4). 42 genes show significant differential expression [*P* < 0.01, lfc > log_2_(2)]. Light points represent genes overexpressed in yellow morph (13 genes), dark points those genes overexpressed in the dark morph (29 genes). Grey points indicate genes that do not show significant differential expression. Red circles indicate genes with a reported link to pigmentation, Blue circles indicate genes involved in nervous system function (see Tables 1 and 2 for references).

To identify genes that are associated with the coloration differences between yellow and dark morph, we screened for differentially expressed genes using RNA-sequencing (RNA-seq) of the integument of yellow (n = 4) and dark morph (n = 4) individuals. Our comparative transcriptomic approach identified 42 differentially expressed genes: 13 genes were expressed at a significantly higher level in the integument of the yellow morph and 29 genes had significantly higher expression in integument of the dark morph (*P* < 0.01, lfc > log_2_(2); Fig. 4; Tables 1, 2). Surprisingly, among those were few genes that had been previously implicated in teleost pigmentation (6 of 42), and all of them were among the genes that were higher expressed in the integument of the yellow morph (Fig. 4; Table 2). Those six pigmentation genes were *hydroxy-delta-5-steroid dehydrogenase, 3 beta- and steroid delta-isomerase 1* (*hsd3b1*)^47^, pteridine biosynthesis enzyme *GTP cyclohydrolase 2* (*gch2*)^48^, carotenoid droplets disperser *perilipin 6* (*plin6*)^49^, melanophore-linage cell marker *microphthalmia-associated transcription factor a* (*mitfa*)^50–52^, *tetratricopeptide repeat protein 39B* (*ttc39b*)^53–55^ and oncogenic transcription factor *forkhead box Q1* (*foxq1a*)^56^.

**Table 1:** List of up-regulated genes in skin of dark morph.

ID	Gene name	Description	Log_2_ FC	log_10_ (*P*)	Process
ENSMZEG00005002732	*snap25a*	Synaptosomal-associated protein 25a	6.57	10.76	Neuronal^57^
ENSMZEG00005013660	*gpm6ab*	Neuronal membrane glycoprotein M6-a	5.21	10.76	Neuronal^58,59^
ENSMZEG00005005539	*snap25b*	Synaptosomal-associated protein 25b	7.21	6.75	Neuronal^57^
ENSMZEG00005020450	*gdf6*	Growth/differentiation factor 6-B	4.86	6.75	–
ENSMZEG00005003539	*vsnl1a*	Visinin-like protein 1a	3.46	6.73	Neuronal^60^
ENSMZEG00005014823	*stxbp6l*	Syntaxin-binding protein 6-like	7.58	6.03	–
ENSMZEG00005025841	*NA*	NA	7.91	6.00	–
ENSMZEG00005027303	*napb*	Beta-soluble NSF attachment protein	2.40	5.54	Neuronal^108,109^
ENSMZEG00005018814	*nsfa*	N-ethylmaleimide-sensitive factor a (vesicle-fusing ATPase)	7.29	5.08	Neuronal^109^
ENSMZEG00005001660	*syt1a*	Synaptotagmin 1a	5.09	4.79	Neuronal^108,110^
ENSMZEG00005009208	*sncb*	Synuclein beta	5.08	4.52	Neuronal^111^
ENSMZEG00005010543	*sez6l2*	Seizure related 6 homolog like 2	7.08	4.37	Neuronal^112^
ENSMZEG00005002426	*nsg2*	Neuronal vesicle trafficking associated 2	6.65	4.30	Neuronal^113,114^
ENSMZEG00005017078	*zbtb20*	Zinc finger and BTB domain containing 20	1.84	4.29	Neuronal^115^
ENSMZEG00005006423	*fabp7*	Fatty acid binding protein 7	6.99	4.29	Neuronal^116^
ENSMZEG00005010741	*slc17a7b*	Solute carrier family 17 (vesicular glutamate transporter), member 7a	6.96	4.25	Neuronal^117,118^
ENSMZEG00005020088	*scg2b*	Secretogranin 2	6.22	4.19	Neuronal^119^
ENSMZEG00005020108	*adcyap1b*	Adenylate cyclase activating polypeptide 1b	2.07	3.82	Neuronal^120^
ENSMZEG00005018886	*gad2*	Glutamate decarboxylase 2	5.96	3.54	Neuronal^121–123^
ENSMZEG00005022306	*tuba1b*	Tubulin alpha-1B chain	3.14	3.20	–
ENSMZEG00005001648	*zgc:65851*	Low molecular weight neuronal intermediate filament	6.68	3.19	Neuronal^124^
ENSMZEG00005017787	*eno2*	Enolase 2	5.77	3.00	Neuronal^125,126^
ENSMZEG00005013731	*slc6a1b*	Solute carrier family 6 (neurotransmitter transporter), member 1b	6.03	2.80	Neuronal^127^
ENSMZEG00005025554	*ncan*	Neurocan core protein	2.51	2.74	Neuronal^128^
ENSMZEG00005025994	*atp1a3a*	Sodium/potassium-transporting ATPase subunit alpha-3	1.42	2.38	Neuronal^129^
ENSMZEG00005014171	*p2ry6*	Pyrimidinergic receptor P2Y6	1.21	2.29	–
ENSMZEG00005015980	*map1aa*	Microtubule associated protein 1A	1.91	2.29	Neuronal^130,131^
ENSMZEG00005012827	*vsnl1*	Visinin-like protein 1	3.98	2.27	Neuronal^60^
ENSMZEG00005025734	NA	NA	3.83	2.06	–

**Table 2:** List of up-regulated genes in skin of yellow morph.

ID	Gene name	Description	Log_2_ FC	log_10_ (*P*)	Process
ENSMZEG00005025109	*foxq1a*	Forkhead box protein Q1	1.99	2.29	Pigmentation^56^
ENSMZEG00005008428	*tmem130*	Transmembrane protein 130	1.77	2.30	–
ENSMZEG00005021959	*rdh11*	Retinol dehydrogenase 11	1.69	2.38	–
ENSMZEG00005002482	*gch2*	GTP cyclohydrolase 2	2.94	2.50	Pigmentation^48^
ENSMZEG00005008959	*mitfa*	Microphthalmia-associated transcription factor	1.15	3.14	Pigmentation^50–52^
ENSMZEG00005002836	*map7a*	Ensconsin	1.11	3.18	–
ENSMZEG00005012260	*scarb1*	Scavenger receptor class B member 1	1.07	3.18	–
ENSMZEG00005020078	*dhrs12*	Dehydrogenase/reductase SDR family member 12	1.90	3.18	–
ENSMZEG00005017109	*plin6*	Perilipin 6	1.94	3.62	Pigmentation^49^
ENSMZEG00005004808	*bscl2*	Seipin	1.96	3.95	–
ENSMZEG00005014299	*mmel1*	Membrane Metalloendopeptidase like 1	2.70	4.17	–
ENSMZEG00005019551	*ttc39b*	Tetratricopeptide repeat protein 39B	2.27	4.30	Pigmentation^53–55^
ENSMZEG00005019286	*hsd3b1*	Hydroxy-delta-5-steroid dehydrogenase, 3 beta- and steroid delta-isomerase 1	1.94	4.43	Pigmentation^47^

In the integument of the dark morph we found no pigmentation genes with significantly higher expression, instead a high number (80% of the genes) of neuronal genes—23 of 29 based on a literature search (Fig. 4; Table 1). Two GO-terms were significantly enriched (p_adj_ < 0.05), syntaxin-1 binding (GO:0017075) and SNARE binding (GO:0000149), both are important protein interactions involved in the regulation of synaptic vesicles at the presynaptic zone of neuronal synapses. Among the most significantly differentially expressed genes are the *synaptosomal-associated protein 25* (*snap25a* and *snap25b*) that is required for the fusion of synaptic vesicles with the presynaptic membrane^57^, the *neuronal membrane glycoprotein M6a* (*gpm6ab*) that is involved in neurite outgrowth and synaptogenesis^58,59^ and the calcium sensor protein *visinin-like protein 1* (*vsnl1a*) that is important for neuronal signaling^60^. The up-regulation of neural and synapse-related genes in the integument suggests differences in neural innervation of the integument that—besides the difference in pigmentation—distinguish the yellow and dark morph of *M. auratus*. We will elaborate in the discussion how this unexpected link between coloration phenotype and neural innervation might be explained.

### Axonal innervation of the scale epithelium is increased in the dark morph of *M. auratus*

**Figure 5:**
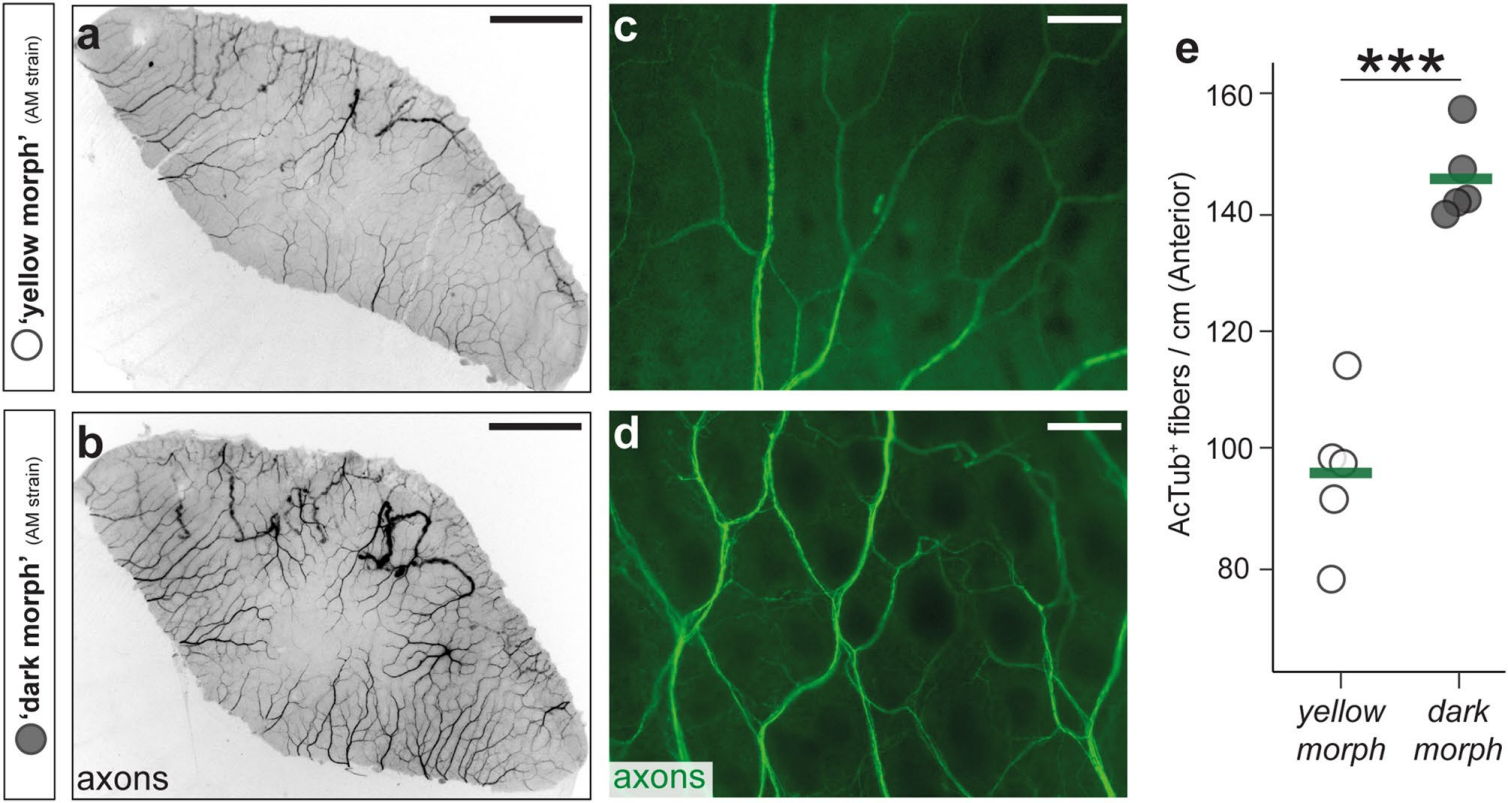
Increased axon innervation in the epidermis of the dark morph. (**a–d**) Immunohistochemistry with Anti-Acetylated Tubulin antibody labeling axonal fibers shows increased innervation of scales of the dark morph/‘dark morph’ (**b**,**d**) compared to the yellow morph/‘yellow morph’ (**a**,**c**). Scale bars are 500 μm in (**a**,**b**) and 50 μm in (**c**,**d**). The complete analysis can be found in Supplementary Fig. S8 (**e**) Axon density on scales is quantified based on Acetylated Tubulin staining. Difference between morphs was evaluated by two-tailed *t* test, n = 5 (individual points). Each individual point represents the mean value of 15 measurement for one fish. Green bars indicate the means. Significant sign: ***P < 0.001.

To find further support for the hypothesis that neural innervation is indeed increased in the dark morph, we performed immunofluorescence staining of axonal fibers on scales of both morphs^61^. We dissected scales from three homologous dorsal–ventral positions and counted the number of nerve fibers (Fig. 5, Supplementary Figs. S6, S7, see details in the methods section). Axons were visualized using Anti-Acetylated Tubulin antibody (AcTub). In accordance with the transcriptome data, we indeed found more AcTub^+^ axons on scales of the dark morph compared to scales of the yellow morph, independent of dorso-ventral level and position on the scale (all *P* < 0.01, two-tailed *t* test, 1.3 to 2-fold change; Fig. 5e, Supplementary Figs. S6, S7, Supplementary Tables S3, S4). Yet, we were concerned that the lower number of axon counts might be also linked to the increased number of melanophores that could mask the fluorescence and thereby result in an underestimation of axons in the dark morph (Supplementary Fig. S8). Therefore, we used a previously described (spontaneous) amelanistic mutant line that lacks all melanic pigmentation due to a loss of the second exon of *oculocutaneous albinism II* (*oca2*) gene^18^. Still, despite this loss-of-function mutation, melanophores seem to be present in both, the yellow and dark morph of the amelanistic line as previously described^18^ (as the morph names are rather confusing descriptions in the amelanistic strain we use quotations marks, i.e. ‘yellow morph’ and ‘dark morph’). Comparably to the wildtype-like strain of *M. auratus*, ‘dark morph’ individuals of the amelanistic *M. auratus* had significantly higher axon density than ‘yellow morph’ individuals (all *P* < 0.01, two-tailed *t* test, 1.3 to 1.8 fold change) (Fig. 5a–d, Supplementary Figs. S6, S7, Tables S3, S4). Lastly, as mentioned in the introduction and discussed below it is dominant males that undergo the color transition and transition into the dark morph, while this has not been reported (or only very rarely) for female individuals. It is therefore possible that the differences in axon density are linked to sex and not to morph. To test this possibility, we performed the same quantifications on scales of a closely related species without color change, *Pseudotropheus cyaneorhabdos* (previously *Melanochromis cyaneorhabdos*), and compared males and females. No significant differences in axon density could be detected (all *P* > 0.05, two-tailed *t* test) (Supplementary Figure S6, S7, Supplementary Tables S3, S4) suggesting that the difference in neural innervation is linked to color morph, but not sex.

## Discussion

In this study, we investigated the cellular and transcriptional differences between the yellow and dark morph of *M. auratus*, a cichlid fish species that is endemic to Lake Malawi^62,63^. Specifically, we asked how the morphological differences can be explained (a) by changes in chromatophore number (b) chromatophore characteristics, (c) multi-dimensional arrangement of chromatophores in the integument and (d) if we find transcriptional signatures that provide us with more mechanistic insights into genetic and cellular correlates as well as the genetic pathways that might be involved in triggering the morphological color change from yellow to dark morph.

Our analysis reveals substantial variation between yellow and dark morph (Fig. 1) ranging from transcriptomic differences, to differences in chromatophore morphology, number and three-dimensional organization as well as—unexpectedly—changes in neural innervation of the integument. These dramatic changes must be occurring during the morphological color change of dominant males that transition over the course of two weeks from the yellow to the dark morph (visual inspection on > 10 individuals). Below we therefore (i) comprehensively discuss the differences between the yellow and the dark morph, (ii) speculate what changes cause the transition and/or what might be merely indirect consequences of the morphological color change.

Our study demonstrates that yellow and dark morph differ greatly on different levels of biological organization ranging from gene expression differences to cellular and ultracellular characteristics of chromatophores as well as cellular composition in the three layers of the integument and on scales. The first observation is that the color of phenotypically similar regions is formed through similar mechanisms (Figs. 2 and 3): The dark stripes of the yellow morph (MLS and DLS) and the dark ventral area and interstripe (vVEN, dVEN and INT) of the dark morph are characterized by a high density of dispersed melanophores (Fig. 2g–i). In contrast, the yellow belly region of the yellow morph (vVEN) has a very low density of melanophores, but high density of large, dispersed xanthophores. A further peculiarity are thick layers of horizontally oriented iridiosomes within the stratum compactum, the deepest layer of the integument. These likely simply serve as a mirror and directly reflect light and thereby intensify the yellow coloration of the overlaying xanthophores. Such mirror-like function of iridosomes have been previously described, i.e. in the japanese Koi fish (*Cyprinus carpio*), where the reflectance is however generated through a fully random organization of iridosomes^64^. Lastly, there are several regions with iridescent white (i.e. INT in yellow morph) to blue (MLS in dark morph) coloration. The major differences to the other regions is (beside the lower density of xanthophores and melanophores) that iridosomes are not oriented horizontally but in an acute angle of about 25° to 50° (Fig. 3r). Such regularly oriented, tilted iridosomes have been previously described in the neon tetra *Paracheirodon innesi*, that is characterized by a single iridescent blue horizontal stripe. The periodically arranged iridosomes constitute a colour-producing microstructure that might cause the blue coloration through multilayer optical interference^45,46^.

At the transcriptomic level we find several pigmentation genes that are differentially expressed. Two of the genes that show higher expression in the yellow morph are *gch2* and *plin6* (Fig. 4; Table 2)., which have been associated with carotenoid and pteridine synthesis—the yellow to orange pigments of xanthophores The Plin6 protein targets the surface of carotenoid droplets and mediates carotenoid droplet dispersion in xanthophores^49^. The gene *gch2* encoding a pivotal protein in the pteridine biosynthetic pathway and is required for xanthophore pigmentation in zebrafish^48^. Also *hsd3b1* and *ttc39b* have been linked to lipid and carotenoid synthesis as well as carotenoid-based coloration differences in cichlids^47,53–55^. Compared to the dark morph, xanthophores are numerous in the yellow morph and densely filled with xanthosomes as supported by light microscopy (Fig. 2) and TEM images (Fig. 3), therefore these transcriptional differences are compatible with the morphological differences. We also find two pigmentation genes that have been associated with melanophore/melanocyte differentiation: *foxq1* and *mitfa*^50–52,56^. As melanophore numbers are lower in the yellow morph, especially in vVEN (Figs. 2 and 3), it is possible that the high expression of these genes indicates that large reservoirs of undifferentiated melanoblasts exist, that only differentiate when the color change is initiated. What remains unclear is, how the melanoblasts are mechanistically kept in an undifferentiated state. Interestingly, several of the other differentially expressed genes have been previously linked to stem cell maintenance including retinol dehydrogenases^65^, *ensconsin/map7a*^66^, *scavenger receptor class B member 1*^67^ and *seipin/bscl2*^68^.

The most striking observation regarding those genes being overexpressed in the dark morph is that a substantial part of these genes (80%) are related to neuronal processes, particularly to synaptogenesis (Fig. 4; Table 1). This observation is further substantiated by immunohistochemistry for the axonal marker acetylated tubulin that demonstrate that there is a substantially higher number of neuronal fibers in scales of the dark morph than in the yellow morph (1.3 to 2-fold change; Fig. 5) across the whole trunk.

To understand the—at first surprising—link between coloration and nervous innervation, there are two facts to consider: Firstly, adult pigment cells derive from postembryonic progenitors that are located in proximity of the dorsal root ganglia. From there these progenitors migrate along peripheral nerves through the myosepta to the skin. Therefore, there is a strong developmental link between differentiation of adult pigment cells and nerve fibers^69–71^. Secondly, the connection between pigment cell remains even after differentiation. Especially melanophores, but also xanthophores and iridophores are controlled by the nervous system that can trigger the aggregation of melanosomes or xanthosomes, as well as reorientation of iridosomes^11,72,73^. These fast intracellular changes trigger rapid *physiological* color change^72^. But neural innervation has been also shown to induce more long-lasting changes (i.e. morphological color change) by triggering remodeling of the cytoskeleton^74^, apoptosis of chromatophores^74,75^, as well as proliferation of chromatophores and changes in pigment synthesis^76^. Very recently, increased neural signaling has been even linked to the depletion of melanocyte stem cell pools leading to hair greying in humans^77^, which supports an evolutionary conserved link between the nervous system and pigment cells.

The morphological color change of *M. auratus* is particularly remarkable because several, oppositely directed changes occur in different parts of the skin (Fig. 2). While in the stripes melanophores are reduced and xanthophores are increased in number, we observe the opposite trend in the interstripe and ventral skin. Additionally, we find increased numbers of iridophores, mainly in the dorsal areas, as well as reorganization of iridosomes, in particular in the MLS. The molecular processes that orchestrate such a complex rearrangement are therefore likely complex itself. Based on our results, we assume that two different processes are occurring: First, we observe changes at the level of pigment cell progenitors, either within the skin or at the postembryonic progenitor niche in vicinity of the dorsal root ganglia that lead to the generation of new chromatophores. As cell–cell interactions are an important factor for color pattern formation in fish, it is possible that these changes do not occur everywhere at the same time, but are propagated across the skin^78^. Recently it has been shown that long-distance signaling relayed by macrophages trigger tissue color pattern remodeling during postembryonic development^79^, so similar processes could certainly play a role in this instance of morphological color change. Some of the genes overexpressed in the yellow morph indicate that these fish might already have a reservoir of melanoblasts that could—triggered by an endocrine, paracrine or neuronal signal—differentiate and thereby realize the morphological color change from the yellow to the dark morph. Second, the overexpression of neural genes and increased number of neural innervations could be an indication for two processes: The nerve fibers could lead to a stronger neural control of chromatophores (i.e. physiological color change). This interpretation is supported by the fact that the melanic patterns do not fade or enhance very much in the yellow morph, while the dark morph shows substantial physiological color change with the dark melanic parts changing from light grey to black and vice versa. Second, it is also possible that the neural fibers have an instructive role and initiate the color change by promoting migration, differentiation of chromatophore progenitors, cell death as well as changes in pigment production or translocation, as shown in other organisms^74–77^.

One central question is how the color change of the different regions (i.e. DLS, INT, MLS, dVEN and vVEN) is orchestrated. As discussed above, changes happen in almost opposite directions, with DLS and MLS becoming lighter (i.e. grey or blue) and INT, dVEN and vVEN becoming darker (i.e. dark grey or black). However, we do not see variation in neural innervation across the dorsoventral axis that could explain these oppositely directed changes and therefore the phenotype remains difficult to explain solely by the innervation. It is possible that this is achieved by a combination of the neuronal trigger and the number of chromatophore precursors that are present in the different regions. Therefore, although our morphological and transcriptomic analyses provide important and novel insights into the molecular and cellular correlates of the color change in *M. auratus*, our analyses cannot indicate whether the nervous system and/or particular genes act directly or indirectly on the distribution, shape and properties of chromatophores and if they are causally involved. An even more detailed analysis will be needed that dissects the molecular and cellular changes in the different skin regions—also at different time points of the transition process—to completely understand this remarkable color change phenotype.

In conclusion, our work provides an integrative and comprehensive description of the transcriptomic, ultrastructural, cellular and neural changes that define the morphological color change of *M. auratus*. It provides therefore the first insights into the unexpectedly massive changes occurring at different levels of biological organization ranging from gene expression changes, to reorientation of reflecting platelets (iridosomes) in iridophores, to changes in chromatophore density and morphology, and changes in neuronal innervation. The color change *M. auratus* was described as “perhaps most remarkable case” of “color change reported for any cichlid” almost 50 years ago^26^—the complexity of the changes that shape and accompany this transition and its evolution are in no way less remarkable.

## Methods

### Animals

Animals used in this study were raised and kept in the animal research facility of the University of Konstanz. All animal samples used in this study were collected in accordance with relevant guidelines and regulations and sampling was approved by the authorities (Regierungspräsidium Freiburg, Anzeige T-16/13).

### Light microscope image acquisition for fish scales

To quantify the change in chromatophore quantities and properties during color transition of *M. auratus*, we measured the coverage, density and dispersed diameter of chromatophores in scales from five homologous pigmented regions in dorsal–ventral axis. In *M. auratus*, chromatophores could be found in multiple layers through the integumentary system and overlaid/underlaid each other. The chromatophores only cover the posterior part of the scales and are arranged in a single layer, which makes the robust chromatophore quantification possible. Five individuals of each color morph were examined for scale chromatophore quantification. Fifteen scales from three dorsal–ventral rows (5 scales each row) were removed from each fish. Scales from most dorsal row covering DLS and INT (Fig. 2a,d); scales from middle row covering MLS and dVEN (Fig. 2b,e); the scales from most ventral row (vVEN) (Fig. 2c,f). Once being removed from the fish, scales were rinsed by Phosphate Buffered Saline (PBS, pH 7.4) and adhered to HistoBond + adhesive microscope slides (Paul Marienfeld). Photographs of *M. auratus* scales were captured on Leica DM6B upright microscope with a Leica DMC 2900 camera. We captured images in three different modes: brightfield for melanophore coverage and dispersed diameter, polarized light for iridophore coverage^7^, fluorescent with GFP filter for xanthophore coverage and dispersed diameter^80,81^. To count the number of melanophores and xanthophores, we treated the scales immediately after taking the above photos with 10 mg/ml adrenaline (SIGMA-ALDRICH) for 5 min at room temperature on microscope slides to aggregate the melanosomes which permit robust cell number quantification (Supplementary Fig. S1). After adrenaline treatment, microscope-slide-adhered scales were rinsed by PBS and photographed using brightfield mode. Leica Application Suite X software was used to capture the photos using the same setting.

### Image and data analysis of scale chromatophores

All light microscope photos were analyzed with Fiji^82^. In order to obtain reliable chromatophore coverage quantification, we manually adjusted the color threshold of the brightfield photos for melanophores, polarized light photos for iridophore and fluorescent photos for xanthophore from non-treated scales (Supplementary Fig. S1). Following this setting, we executed the “Analyze Particles” function to obtain all three chromatophore coverage. For chromatophore size measurement, we randomly selected 10 melanophores from brightfield photos and 10 randomly xanthophores from fluorescent photos for each pigmented region (DLS, INT, MLS, dVEN, vVEN) and measured the dispersed diameter of pigment covered parts of the chromatophores. The number of melanophores and xanthophores was counted from the same scales after the epinephrine treatment. Although we could identify iridophores and quantified the iridophore coverage in all pigmented regions before and after epinephrine treatment (Supplementary Fig. S1), the boundaries of each iridophore could not be identified. Therefore, we were unable to quantify the iridophore number and measure the iridophore size.

### Transmission electron microscope image acquisition for fish integument

Melanic and non-melanic skin with scales attached were dissected in buffer pre-fixative (2% formaldehyde, freshly depolymerized from paraformaldehyde (Merch), 2.5% glutardialdehyde (Agar Scientific) in 0.1 M HEPES (AppliChem) with 5 mM MaCl_2_, 5 mM CaCl_2_, 0.125 mM MgSO_4_ and 0.3 M sucrose for osmolarity, pH 7.5) and fixed in fresh-made, pre-cooled fixative (same as buffer pre-fixative) at 0 °C for 2 h. After osmification in 2% OsO_4_ (SERVA) (buffered with HEPES, pH 7.5) at 0 °C for 2 h, samples were pre-dehydrated in 30% and 50% Ethanol and en-bloc stained the samples by 2% uranyl acetate (Merck) in 70% Ethanol. We dehydrated the samples in graded series of acetone solutions and embedded in SPURR (TedPella) (ERL 4,206, DER 736, NSA, DMAE). 50 nm ultra-thin sections were cut by a Leica Ultracut microtome with an ultra 45° knife (Diatome, Switzerland). Sections were contrasted with uranyl acetate and lead citrate before examined with a Zeiss TEM 912. Images were processed with Fiji and stitched with TrackEM2^83^. Iridosome orientation was estimated by measuring the angle between iridophore and skin surface. Iridosome orientation, thickness and distance between iridosomes, as well as melanosome density were analyzed with Fiji^82^. Plots in Fig. 3q–t and Supplementary Figs S2. S3 and S4 were generated using ggplot^84^ in the R-environment^85^ and assembled and annotated in Adobe Illustrator v.24.0.3 (Adobe Systems Software). Illustration of the chromatophore arrangement in the integument (Supplementary Fig S5) was generated using by Adobe Illustrator v.24.0.3 (Adobe Systems Software).

### RNA extraction and purification

RNA extraction and purification were performed as previously described^86^. Briefly, dissected skin samples of eight individuals (four for each morph) were kept in RNAlater (Invitrogen) at − 20 °C. RNAlater was removed prior to homogenization. Skin samples and the appropriate amount of TRIzol (Invitrogen) (1 ml TRIzol per 100 mg sample) were homogenized in 2 ml Lysing Matrix A tube (MP Biomedicals) using FastPrep-24 Classic Instrument (MP Biomedicals). Subsequent purification and DNase treatment were performed with RNeasy Mini Kit (Qiagen) and RNase-Free DNase Set (Qiagen). Following extraction and purification, RNA was quantified using the Qubit RNA HS Assay Kit (Invitrogen) with a Qubit Fluorometer (Life Technologies). The RNA integrity number (RIN) was checked using an RNA 6000 Pico Kit (Agilent) on a 2100 Bioanalyzer System (Agilent).

### RNA library preparation and sequencing

RNA-seq libraries were prepared using the TruSeq Stranded mRNA Library Prep Kit (Illumina) according to the manufacturer’s protocol. Briefly, 1 μg RNA was put into mRNA selection by poly-T oligo attached magnetic beads followed by fragmentation (94 °C for 6 min). The cleaved mRNA fragments were reverse transcribed into first-strand cDNA using GoScript Reverse Transcriptase (Promega) and random hexamer primers (Illumina). We used Illumina-supplied consumables to synthesize second-strand cDNA following by adenylating 3′ ends. Barcoded adapters from TruSeq RNA CD Index Plate (Illumina) were ligated to the ends of the double-strand cDNA. The final libraries were amplified using 15 PCR cycles and quantified and quality-assessed using an Agilent DNA 12000 Kit on a 2100 Bioanalyzer (Agilent). Indexed DNA libraries were normalized. Libraries were sequenced on a HiSeq X Ten platform (BGI Genomics, Beijing).

### Mapping and data processing

For adapter trimming trimmomatic 0.38 was used^87^. The processed raw reads (mean: 41.7 million reads per sample) were aligned to the *Maylandia zebra* genome (M_zebra_UMD2a, INSDC Assembly GCA_000238955.5, Apr 2018) using the STAR RNA-seq aligner (version 2.6.1d)^88^. *Maylandia zebra* and *M. auratus* are very closely related, why this genome was used as a reference. Expression counts were calculated with RSEM^89^ using the ENSEMBL annotation. Quality control was done using MultiQC ^90^. 89% of the reads were uniquely mapped. The short-read data has been archived in NCBI SRA database under the Bioproject accession number PRJNA635556 (SRR11862158–SRR11862165).

### Data analysis

The data was analysed in R using the DESEQ2 1.22.1 pipeline^91^ in R^92^. The following packages were additionally used: BiocParallel 1.16.2^93^, tximport 1.10.1^94^, stringR 1.4.0^95^, edgeR 3.24.1^96^, vsn 3.50.0^97^, ggplot2 3.1.1^98^, RColorBrewer 1.1–2^99^, vidger 1.2.0^100^, pheatmap 1.0.12^101^, cowplot 0.9.4^102^ and dplyr 0.8.0.1^103^. GO term analyses was performed by extracting GO terms for the *M. zebra* ENSEMBL genome annotation using biomaRt 2.38.0^104^. Next, we used goseq 1.34.1^105^ to correct for transcript length correction and selection-unbiased testing for category enrichment amongst differentially expressed genes. P-values were adjusted with Benjamini & Hochberg correction.

### Axon staining and density quantification on scales

As the RNA-seq result showing that the expression of neural genes is upregulated in the dark morph, we performed immunofluorescent staining for axon to further substantiate the expression data. Fish scales (15 scales/fish) of a total of 29 fish (5 individuals per morph except for the individuals of the ‘yellow morph’ of the amelanistic individuals for which we only had 4 individuals) were removed from the homologous regions for chromatophores measurement (5 scales from each dorsal–ventral row, 3 rows from each fish). Immunostaining was performed based on previously described with modification^106^. Scales were fixed in Dent’s fixative (20% dimethyl sulfoxide (DMSO), 80% Methanol) at 4 °C overnight then kept in 100% Methanol at − 20 °C. We rehydrate the samples by graded series of Methanol/PBS. After several washes in PBS and PBS 0.1% Triton X-100, we permeabilizated the samples with permeabilization buffer (1% normal goat serum (Sigma), 0.4% Triton X-100 in PBS). Scales were then blocked in 10% normal goat serum with 0.4% Triton 100-X in PBS for minimally 2 h and incubated at 4 °C overnight with mouse monoclonal anti-acetylated-tubulin antibody (1:250; clone 6-11B-1; Sigma-Aldrich # T6793) in PBS supplemented with 10% normal goat serum and 0.1% Triton X-100. Although this primary antibody was raised against acetylated tubulin of sea urchin, it has been used for detection of acetylated tubulin from several tissues of many organisms, including fish scales^61,107^. The next day scales were washed by PBS supplemented with 0.1% Tween-20 (PBST) several times and the staining was achieved by incubation with secondary antibody (Goat-anti-Mouse IgG (H + L) SuperClonal Secondary Antibody, Alexa Fluor 488 conjugate, Invitrogen # A28175, 1:400) at 4 °C overnight. The next day scales were washed by PBST and counterstain with 4′,6-Diamidino-2-phenylindole dihydrochloride (DAPI) (1/5000; Sigma) in PBS. We mounted the scales on HistoBond + adhesive microscope slides in Mowiol mounting medium (Sigma). Photographs were taken with a Leica DM6B microscope with a Leica DFC3000G black and white camera.

Axon density was quantified whit Fiji^82^. We quantified the axon density from both anterior and posterior parts of the scales (Fig. 5, Supplementary Figs. S6, S7). For axon density in anterior of scales, the two distinct endpoints of measurement line segment were located in the most dorsal and the most ventral edge of epidermis which indicated by DAPI staining. For axon density in the posterior of scales, the two distinct endpoints of the measurement line segment were located in the most dorsal and the most ventral centiis. Following this setting we executed the “Plot Profile” function and “Find Peaks” function of BAR plugin to obtain the axon density. All statistical tests were performed in R^85^ and plotted using ggplot^84^ in the R-environment^85^.

## Supplementary information

Supplementary file1
