# Supplementary material for: Neural innervation as a potential trigger of morphological color change and sexual dimorphism in cichlid fish
Source: Sci Rep. 2020 Jul 23;10:12329. doi: 10.1038/s41598-020-69239-w (PMC7378239; doi:10.1038/s41598-020-69239-w)
Supplement: Supplementary file 1 — Supplementary file1 [file 41598_2020_69239_MOESM1_ESM.docx]

Supplementary Material

**Neural innervation as a potential trigger of morphological color change and sexual dimorphism in cichlid fish**

Yipeng Liang^1^, Axel Meyer^1*^, Claudius F. Kratochwil^1*^

^1^Zoology and Evolutionary Biology, Department of Biology, University of Konstanz, Universitätsstrasse 10, 78457 Konstanz, Germany

*email: Claudius.Kratochwil@uni-konstanz.de and Axel.Meyer@uni-konstanz.de

**Supplementary text**

**Additional description of chromatophore property differences between yellow and dark morphs of *M. auratus***

Consistent with the strong phenotypic differences in pigmentation (Fig. 1 and Fig. 2a–f), melanophore coverage differed significantly between dark and yellow morphs (all *P* < 0.001, two-tailed *t* test). Coverage was lower in the stripe region of the dark morph [DLS (84.69% in yellow morph to 29.40% in dark morph) and MLS (79.31% to 10.65%)] and higher in the interstripe and belly region of the dark morph [INT (1.34% to 73.24%), dVEN (0.61% to 69.36%), and vVEN (0.22% to 69.88%)] (Fig. 2g, Supplementary Table S1, S2). Consistent with the differences in coverage, melanophore cell density also differed significantly (all *P* < 0.001, two-tailed *t* test) and was lower in stripes of the dark morph [DLS (377.34 cell/mm^2^ to 186.36 cell/mm^2^) and MLS (287.67 cell/mm^2^ to 81.64 cell/mm^2^)] and higher in the interstripe and belly regions [INT (52.83 cell/mm^2^ to 301.71 cell/mm^2^), dVEN (22.93 cell/mm^2^ to 212.81 cell/mm^2^), vVEN (3.49 cell/mm^2^ to 134.70 cell/mm^2^) (Fig. 2h, Supplementary Table S1, S2). The average melanosome dispersal diameter also differed significantly (all *P* < 0.01, two-tailed *t* test) in all five homologous pigment regions and was lower in the stripes of the dark morph [DLS (Ø71.60 µm to Ø49.71 µm) and MLS (Ø79.16 µm to Ø 38.25 µm)] and higher in the interstripe and belly regions of the dark morph [INT (Ø20.56 µm to Ø78.04 µm), dVEN(Ø 17.97 µm to Ø 89.77 µm) and vVEN (Ø11.09 µm to Ø 104.97 µm)] (Fig. 2i, Supplementary Table S1, S2).

Differences in xanthophore coverage were restricted to the ventral regions, where coverage was higher in the yellow morph [1.68% in dVEN of yellow morph to 0.23% in dVEN of dark morph (*P* < 0.05), 11.22% to 0.05% in vVEN (*P* <0.01)]. In the other regions, coverage was similar and did not differ significantly (0.25% to 1.37% in DLS, 1.68% to 0.40% in INT, 0.26% to 0.76% in MLS) (Fig. 2j, Supplementary Table S1, S2). For Xanthophore cell density differences the patterns were similar (DLS, 30.56 cell/mm^2^ to 55.77 cell/mm^2^; MLS, 17.28 cell/mm^2^ to 31.97 cell/mm^2^; INT, 79.86 cell/mm^2^ to 27.81 cell/mm^2^, dVEN, 36.89 cell/mm^2^ to 16.49 cell/mm^2^), however only the ventral belly region showed a significantly higher cell density in the yellow morph (99.29 cell/mm^2^ to 2.38 cell/mm^2^ in vVEN; *P* < 0.01) (Fig. 2k, Supplementary Table S1, S2). Xanthophore size/dispersal was higher in both dVEN and vVEN of the yellow morph (both *P* < 0.001; Ø33.23µm to Ø15.62µm in dVEN, Ø64.61µm to Ø18.18µm in vVEN). In the dorsal regions there was no significant difference (Ø16.74µm to Ø15.85µm in DLS, Ø19.76µm to Ø13.10µm in INT, Ø21.083µm to Ø20.45µm in MLS) between two color morphs (Fig. 2l, Supplementary Table S1, S2).

Although we could identify iridophores by polarized light illumination, we were not able to demarcate individual cells. Therefore, we only measured iridophore coverage but not density and diameter of iridophores. Iridophore coverage increased significantly in the two regions where we observe iridescent white/blue coloration in the dark morph (0.15% to 3.37% in DLS with *P* < 0.01; 0.02% to 0.76% in MLS with *P* < 0.05) (Fig. 2m, Supplementary Table S1, S2). When all data were analyzed by a principal component analysis, we observe that the five homologous regions of the two morphs largely cluster by color (Fig. 2n).

**Supplementary Figures**


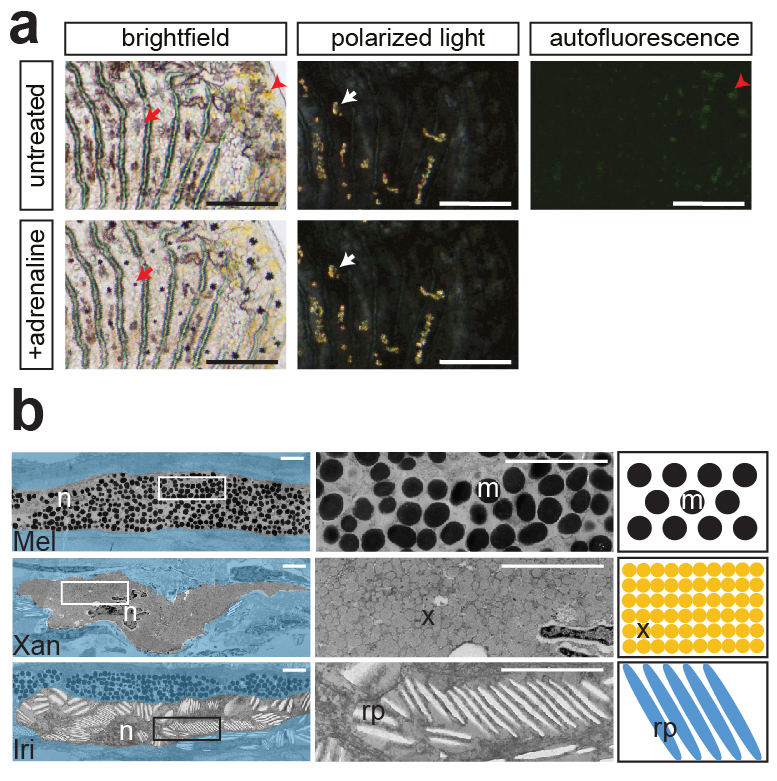


**Supplementary Fig S1. Chromatophores in light microscopical and TEM analysis.**

**(a)** Photographs of same part of a scale under different condition: left panels, brightfield images; middle panels, images under polarized light; right panel, fluorescent image. Upper row, images before adrenaline treatment; lower row, images after adrenaline treatment. Red arrows indicate the same melanophore before and after adrenaline treatment. White arrows indicate the iridophore(s) before and after adrenaline treatment. Red arrowheads indicate the same xanthophore under brightfield and fluorescence. **(b)** Transmission electron microscopy (TEM) images of melanophore (upper row), xanthophore (middle row) and iridophore (lower row). Left panels, low magnification (adjacent cells masked). Middle panels, boxed regions. Right panels, illustrations of chromatophores. Abbreviations: Mel, melanophore; Xan, xanthophore; Iri, iridophore; n, nucleus; m, melanosome; x, xanthosome/pterinosome; rp, reflecting platelet. Scale bars are 20 μm in (a) and 2 μm in (b).


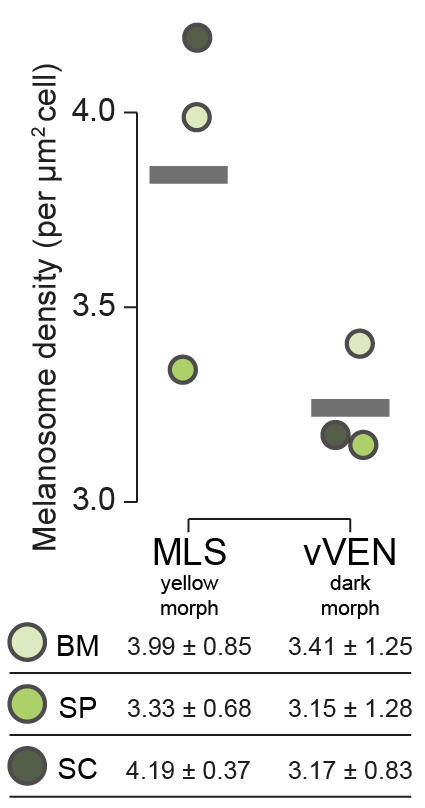


**Supplementary Figure S2. Melanosome density from the black pigmented regions.**

Each indicidual dot indicates the mean valuve from the same layer. Gray bars represent the mean values of all three layers from the same regions. Numbers under the plot: mean ± SD.


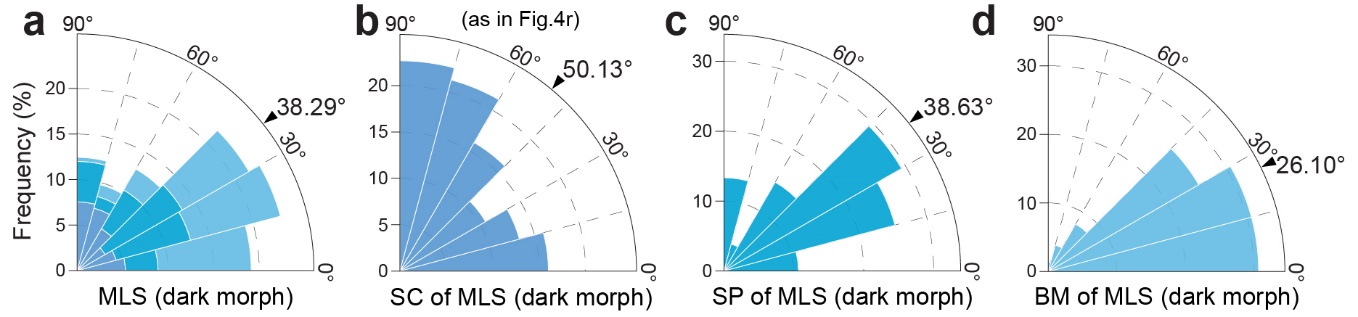


**Supplementary Fig S3. Polar chart of the angle of iridosomes in the MLS of the dark morph.**

Result from all three layers (a), stratum compactum, SC (b), stratum spongiosum, SP (c) and basal membrane, BM (d). Colors in (a) represent results from (b-c).


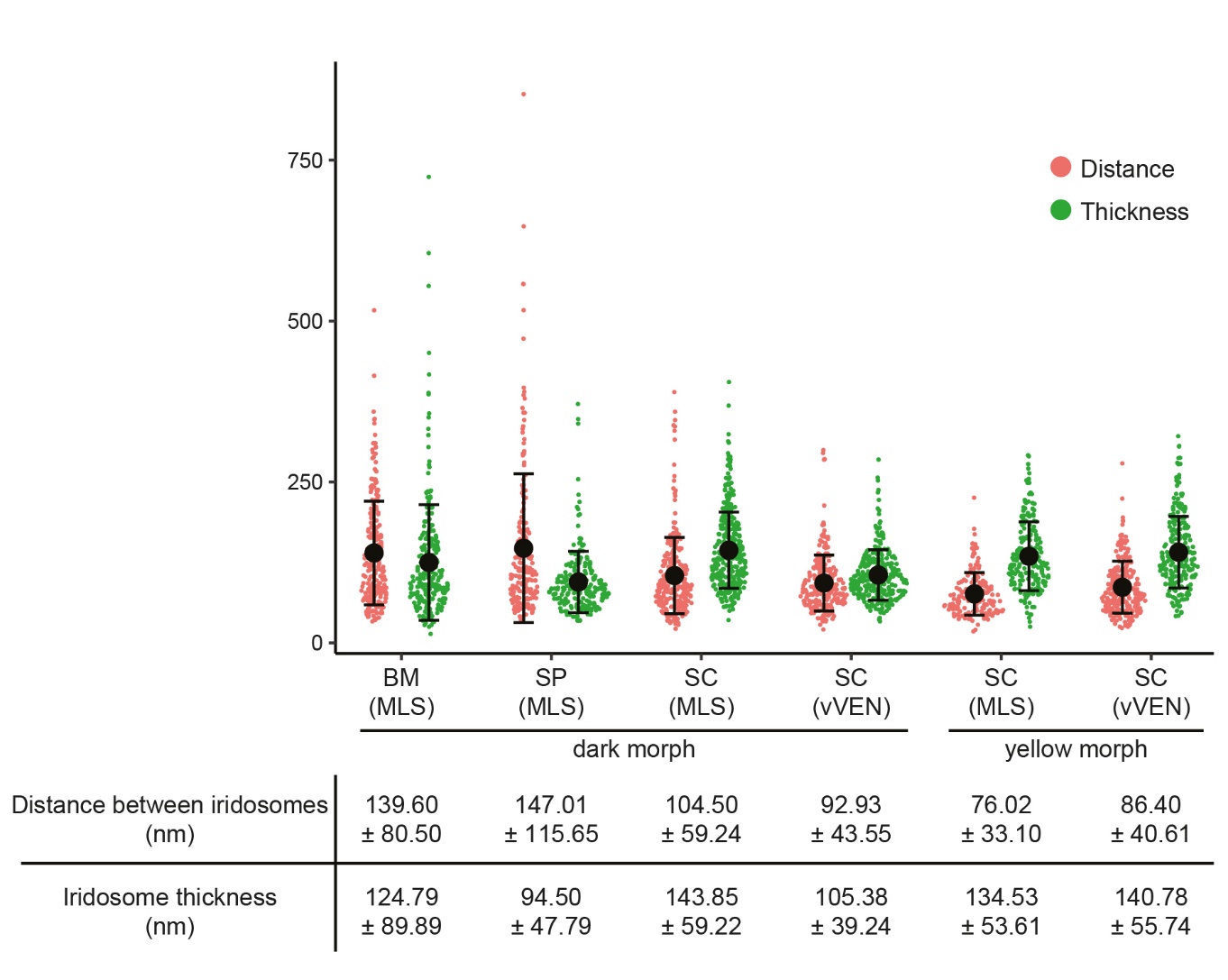


**Supplementary Figure S4. Distance between iridosomes and the thickness of iridosomes.**

Small dots represent the value of individual measurement. Large black dots indicate the mean values from the same layers. Error bars indicated ± SD.


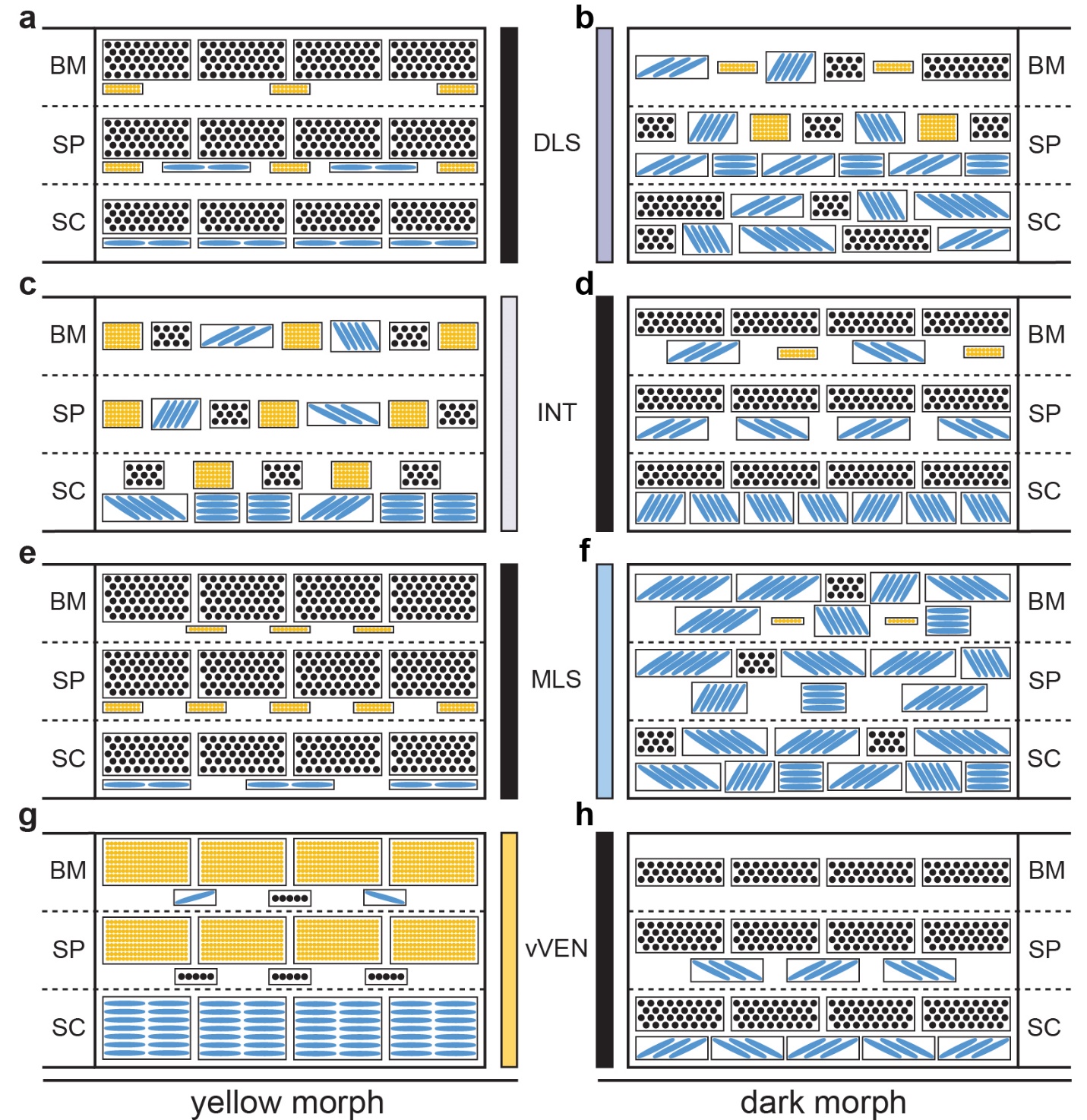


**Supplementary Fig S5. Chromatophores organization in skin of yellow and dark morph *M. auratus*.**

Ultrastructural illustration of chromatophore arrangement in integument: DLS (a), INT (c), MLS (e) and vVEN (g) in yellow morph *M. auratus*; DLS (b), INT (d), MLS (f) and vVEN (h) in dark morph *M. auratus*. Illustrations of chromatophores are based on Supplementary Fig S1b: Black dots represent melanosomes, yellow dots repersent xanthosomes and blue platelets represent iridosomes/reflecting platelets.


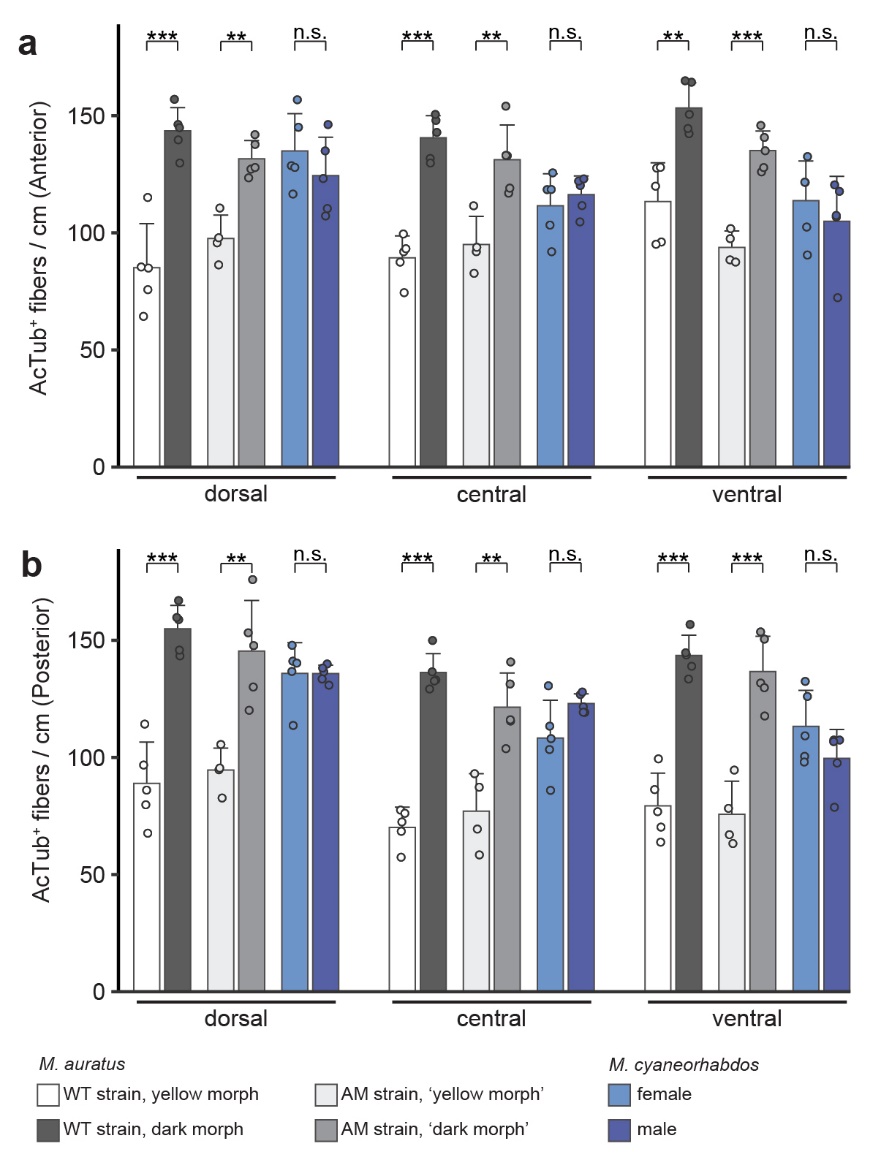


**Supplementary Fig. S6. Axon density on scales across dosal-ventral axis.**

Each point represents mean value of 5 scales from the same dorsal-ventral position within one individual. Error bars represent +SD. Differences between morphs/sexes in the same species and genotype were evaluated by two-tailed *t* test, n = 4 (*M.auratus* AM ‘yellow morph’) or 5 (*M. auratus* WT yellow morph and dark morph, *M. auratus* AM ‘dark morph’, *M. cyneorhabdos* female and male). Significant sign: *** *P*<0.001, ** *P*<0.01, n.s. non-significant.


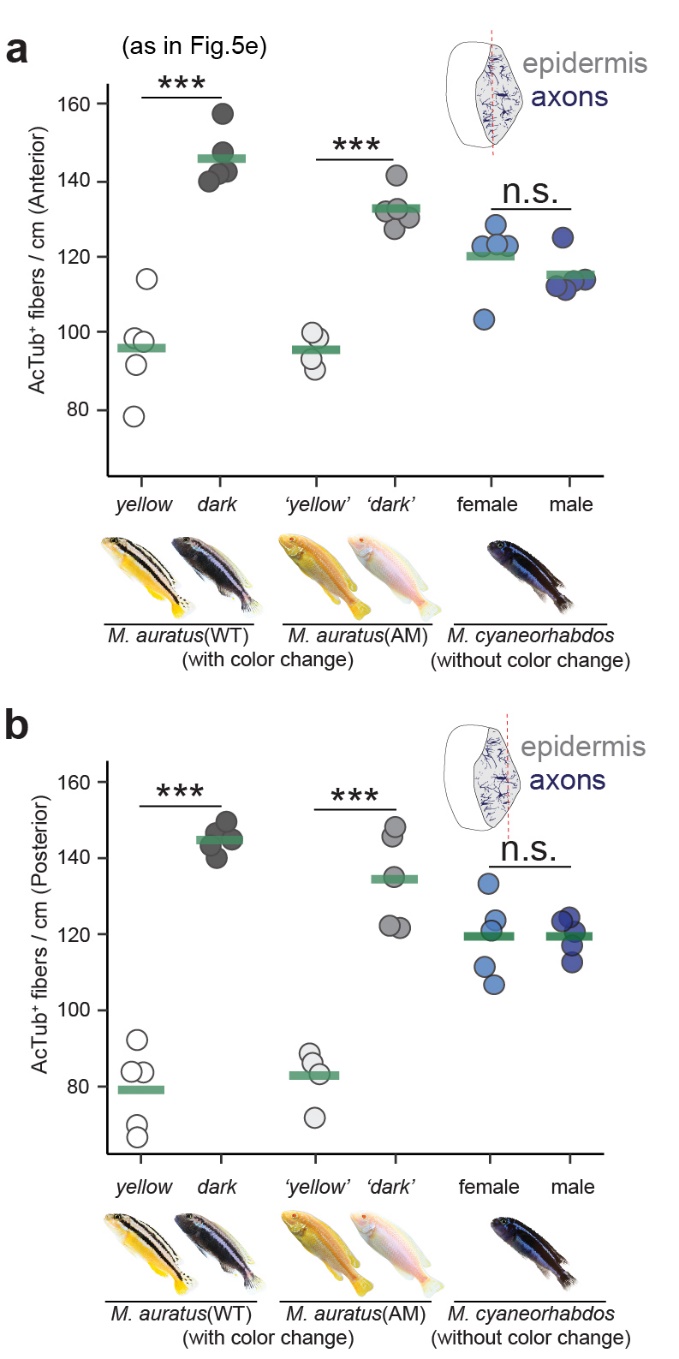


**Supplementary Fig S7. Axon density on scales.**

Axon density on scales is quantified based on Acetylated Tubulin staining. Red dash line crossing the scales in the up-right corner of (a) and (b) indicated the measurement position. **(a)** Axon density in anterior of scales. The two distinct endpoints of measurement are located in the most dorsal and the most ventral edge of epidermis. **(b)** Axon density in posterior of scales. The two distinct endpoints of measurement are located in the most dorsal and the most ventral centiis. Differences between morphs/sexes in the same species and genotype were evaluated by two-tailed *t* test, n = 4 (*M.auratus* AM ‘yellow morph’) or 5 (*M. auratus* WT yellow morph and dark morph, *M. auratus* AM ‘dark morph’, *M. cyneorhabdos* female and male) (individual dots). Each individual dot represents the mean value of measurement for different fishes. Green bars indicate the means. Significant sign: *** *P*<0.001, n.s. non-significant.

**
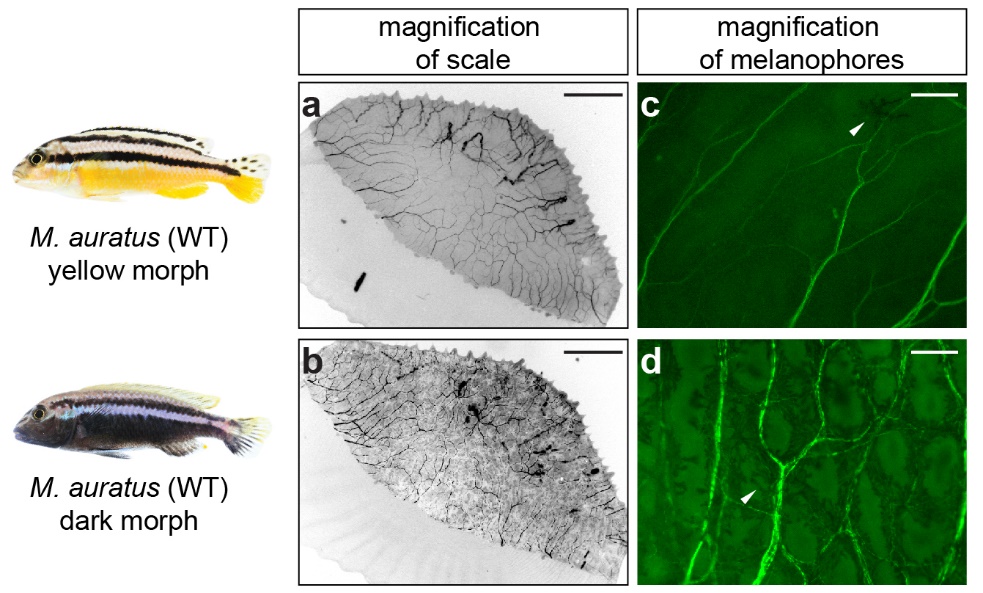
**

**Supplementary Fig S8. Immunofluorescence staining of axon in wild-type *M. auratus*.**

**(a, b)** Representative images of Acetylated Tubulin staining labeled scales from yellow morph and dark morph *M. auratus*. **(c, d)** High magnification microscope photographs of Acetylated Tubulin staining on scales. Melanophores can be observed in wild-type (WT) yellow morph and dark morph (white arrowhead in c and d). Scale bars are 500 μm in (a, b), 50 μm in (c, d).

**Supplementary Table S1. Chromatophore measurement and results of the statistical tests (within morph and individual)**

| yellow morph | Melanophore coverage (%) | | | | | ANOVA | | Tukey HSD *P* value | | | | | | | | | |
| --- | --- | --- | --- | --- | --- | --- | --- | --- | --- | --- | --- | --- | --- | --- | --- | --- | --- |
|  | **DLS** | **INT** | **MLS** | **dVEN** | **vVEN** | **F-value** | ***P* value** | **DLS**  **vs.**  **INT** | **DLS**  **vs.**  **dVEN** | **DLS**  **vs.**  **vVEN** | **MLS**  **vs.**  **INT** | **MLS**  **vs.**  **dVEN** | **MLS**  **vs.**  **vVEN** | **DLS**  **vs.**  **MLS** | **INT**  **vs.**  **dVEN** | **INT**  **vs.**  **vVEN** | **dVEN**  **vs.**  **vVEN** |
| Fish 1 | 75.505 ± 12.057 | 0.061 ± 0.068 | 74.516 ± 6.433 | 0.327 ± 0.338 | 0.217 ± 0.405 | 224.4 | <0.001 | <0.001 | <0.001 | <0.001 | <0.001 | <0.001 | <0.001 | 0.999 | 1.000 | 1.000 | 1.000 |
| Fish2 | 82.986 ± 2.933 | 0.138 ± 0.075 | 82.585 ± 4.918 | 0.064 ± 0.055 | 0.000 ± 0.000 | 1565 | <0.001 | <0.001 | <0.001 | <0.001 | <0.001 | <0.001 | <0.001 | 0.999 | 1.000 | 1.000 | 1.000 |
| Fish3 | 82.538 ± 7.863 | 3.659 ± 2.394 | 88.872 ± 2.968 | 1.094 ± 0.959 | 0.430 ± 0.326 | 685.5 | <0.001 | <0.001 | <0.001 | <0.001 | <0.001 | <0.001 | <0.001 | 0.120 | 0.838 | 0.6963 | 0.999 |
| Fish4 | 92.932 ± 0.654 | 1.388 ± 1.026 | 86.080 ± 2.984 | 0.383 ± 0.488 | 0.235 ± 0.168 | 5572 | <0.001 | <0.001 | <0.001 | <0.001 | <0.001 | <0.001 | <0.001 | <0.001 | 0.810 | 0.724 | 1.000 |
| Fish5 | 89.488 ± 4.728 | 1.470 ± 0.928 | 64.514 ± 26.113 | 1.166 ± 0.888 | 0.194 ± 0.196 | 64.23 | <0.001 | <0.001 | <0.001 | <0.001 | <0.001 | <0.001 | <0.001 | 1.000 | 1.000 | 1.000 | 1.000 |
| Average | 84.690 ± 6.761 | 1.343 ± 1.455 | 79.313 ± 9.873 | 0.607 ± 0.493 | 0.215 ± 0.153 | 341 | <0.001 | <0.001 | <0.001 | <0.001 | <0.001 | <0.001 | <0.001 | 0.529 | 0.999 | 0.997 | 1.000 |

| dark morph | Melanophore coverage (%) | | | | | ANOVA | | Tukey HSD *P* value | | | | | | | | | |
| --- | --- | --- | --- | --- | --- | --- | --- | --- | --- | --- | --- | --- | --- | --- | --- | --- | --- |
|  | **DLS** | **INT** | **MLS** | **dVEN** | **vVEN** | **F-value** | ***P* value** | **DLS**  **vs.**  **INT** | **DLS**  **vs.**  **dVEN** | **DLS**  **vs.**  **vVEN** | **MLS**  **vs.**  **INT** | **MLS**  **vs.**  **dVEN** | **MLS**  **vs.**  **vVEN** | **DLS**  **vs.**  **MLS** | **INT**  **vs.**  **dVEN** | **INT**  **vs.**  **vVEN** | **dVEN**  **vs.**  **vVEN** |
| Fish 1 | 37.438 ± 9.177 | 63.585 ± 4.867 | 12.045 ± 1.853 | 61.942 ± 14.072 | 73.720 ± 4.387 | 47.32 | <0.001 | <0.001 | <0.001 | <0.001 | <0.001 | <0.001 | <0.001 | <0.001 | 0.998 | 0.312 | 0.187 |
| Fish2 | 20.253 ± 7.909 | 76.721 ± 2.780 | 1.058 ± 0.851 | 72.239 ± 8.648 | 70.114 ± 9.427 | 129.8 | <0.001 | <0.001 | <0.001 | <0.001 | <0.001 | <0.001 | <0.001 | <0.01 | 0.837 | 0.559 | 0.987 |
| Fish3 | 33.900 ± 16.729 | 66.096 ± 9.741 | 13.650 ± 3.907 | 71.898 ± 8.804 | 68.006 ± 9.786 | 29.3 | <0.001 | <0.001 | <0.001 | <0.001 | <0.001 | <0.001 | <0.001 | <0.05 | 0.907 | 0.998 | 0.977 |
| Fish4 | 21.496 ± 6.534 | 75.135 ± 5.069 | 7.856 ± 4.878 | 58.924 ± 16.734 | 62.465 ± 9.327 | 45.45 | <0.001 | <0.001 | <0.001 | <0.001 | <0.001 | <0.001 | <0.001 | 0.202 | 0.094 | 0.262 | 0.976 |
| Fish5 | 33.900 ± 8.158 | 84.657 ± 4.660 | 18.658 ± 4.670 | 81.777 ± 2.971 | 75.108 ± 5.591 | 153.7 | <0.001 | <0.001 | <0.001 | <0.001 | <0.001 | <0.001 | <0.001 | <0.01 | 0.918 | 0.080 | 0.337 |
| Average | 29.397 ± 7.926 | 73.239 ± 8.519 | 10.653 ± 6.612 | 69.356 ± 9.122 | 69.883 ± 5.016 | 71.34 | <0.001 | <0.001 | <0.001 | <0.001 | <0.001 | <0.001 | <0.001 | <0.01 | 0.925 | 0.954 | 1.000 |

| yellow morph | Melanophore density (cells/mm^2^) | | | | | ANOVA | | Tukey HSD *P* value | | | | | | | | | |
| --- | --- | --- | --- | --- | --- | --- | --- | --- | --- | --- | --- | --- | --- | --- | --- | --- | --- |
|  | **DLS** | **INT** | **MLS** | **dVEN** | **vVEN** | **F-value** | ***P* value** | **DLS**  **vs.**  **INT** | **DLS**  **vs.**  **dVEN** | **DLS**  **vs.**  **vVEN** | **MLS**  **vs.**  **INT** | **MLS**  **vs.**  **dVEN** | **MLS**  **vs.**  **vVEN** | **DLS**  **vs.**  **MLS** | **INT**  **vs.**  **dVEN** | **INT**  **vs.**  **vVEN** | **dVEN**  **vs.**  **vVEN** |
| Fish 1 | 294.61 ± 30.77 | 10.69 ± 7.41 | 225.00 ± 35.93 | 10.34 ± 6.30 | 0.53 ± 1.19 | 211.8 | <0.001 | <0.001 | <0.001 | <0.001 | <0.001 | <0.001 | <0.001 | <0.001 | 1.000 | 0.943 | 0.950 |
| Fish2 | 394.49 ± 38.44 | 26.93 ± 13.41 | 319.55 ± 41.74 | 9.64 ± 3.49 | 0.28 ± 0.62 | 267 | <0.001 | <0.001 | <0.001 | <0.001 | <0.001 | <0.001 | <0.001 | <0.01 | 0.831 | 0.507 | 0.978 |
| Fish3 | 368.99 ± 75.90 | 114.46 ± 20.01 | 292.73 ± 42.36 | 37.83 ± 28.45 | 6.49 ± 1.65 | 72.55 | <0.001 | <0.001 | <0.001 | <0.001 | <0.001 | <0.001 | <0.001 | 0.063 | 0.061 | <0.01 | 0.760 |
| Fish4 | 405.11 ± 60.29 | 50.35 ± 24.27 | 351.29 ± 20.68 | 20.25 ± 7.28 | 5.02 ± 1.58 | 201.9 | <0.001 | <0.001 | <0.001 | <0.001 | <0.001 | <0.001 | <0.001 | 0.078 | 0.543 | 0.175 | 0.932 |
| Fish5 | 423.49 ± 66.17 | 61.71 ± 24.58 | 249.80 ± 48.49 | 36.61 ± 17.65 | 5.16 ± 2.77 | 103.1 | <0.001 | <0.001 | <0.001 | <0.001 | <0.001 | <0.001 | <0.001 | <0.001 | 0.846 | 0.191 | 0.711 |
| Average | 377.34 ± 50.27 | 52.83 ± 39.78 | 287.67 ± 51.11 | 22.93 ± 13.71 | 3.49 ± 2.88 | 106.3 | <0.001 | <0.001 | <0.001 | <0.001 | <0.001 | <0.001 | <0.001 | <0.01 | 0.711 | 0.260 | 0.919 |

| dark morph | Melanophore density (cells/mm^2^) | | | | | ANOVA | | Tukey HSD *P* value | | | | | | | | | |
| --- | --- | --- | --- | --- | --- | --- | --- | --- | --- | --- | --- | --- | --- | --- | --- | --- | --- |
|  | **DLS** | **INT** | **MLS** | **dVEN** | **vVEN** | **F-value** | ***P* value** | **DLS**  **vs.**  **INT** | **DLS**  **vs.**  **dVEN** | **DLS**  **vs.**  **vVEN** | **MLS**  **vs.**  **INT** | **MLS**  **vs.**  **dVEN** | **MLS**  **vs.**  **vVEN** | **DLS**  **vs.**  **MLS** | **INT**  **vs.**  **dVEN** | **INT**  **vs.**  **vVEN** | **dVEN**  **vs.**  **vVEN** |
| Fish 1 | 237.18 ± 38.87 | 257.96 ± 15.65 | 106.44 ± 24.50 | 201.46 ± 21.39 | 132.58 ± 3.99 | 38.02 | <0.001 | 0.646 | 0.163 | <0.001 | <0.001 | <0.001 | 0.435 | <0.001 | <0.01 | <0.001 | <0.01 |
| Fish2 | 132.88 ± 28.66 | 256.31 ± 22.33 | 22.40 ± 5.61 | 173.00 ± 14.85 | 118.64 ± 7.73 | 110.9 | <0.001 | <0.001 | <0.05 | 0.725 | <0.001 | <0.001 | <0.001 | <0.001 | <0.001 | <0.001 | <0.01 |
| Fish3 | 221.41 ± 26.88 | 287.56 ± 27.75 | 145.99 ± 14.04 | 234.90 ± 41.58 | 151.63 ± 19.49 | 23.46 | <0.001 | <0.01 | 0.935 | <0.01 | <0.001 | <0.001 | 0.997 | <0.01 | <0.05 | <0.001 | <0.001 |
| Fish4 | 159.98 ± 36.22 | 403.15 ± 53.63 | 37.31 ± 18.65 | 241.48 ± 33.96 | 143.27 ± 42.49 | 61.93 | <0.001 | <0.001 | <0.05 | 0.958 | <0.001 | <0.001 | <0.01 | <0.001 | <0.001 | <0.001 | <0.01 |
| Fish5 | 180.35 ± 16.94 | 303.57 ± 30.04 | 96.07 ± 3.83 | 213.20± 13.25 | 127.37 ± 10.57 | 109.4 | <0.001 | <0.001 | <0.05 | <0.001 | <0.001 | <0.001 | 0.065 | <0.001 | <0.001 | <0.001 | <0.001 |
| Average | 186.36 ± 43.02 | 301.71 ± 60.14 | 81.64 ± 51.09 | 212.81± 27.49 | 134.70 ± 13.01 | 19.16 | <0.001 | <0.01 | 0.859 | 0.337 | <0.001 | <0.001 | 0.312 | <0.01 | <0.05 | <0.001 | 0.059 |

| yellow morph | Melanophore dispersed diameter (µm) | | | | | ANOVA | | Tukey HSD *P* value | | | | | | | | | |
| --- | --- | --- | --- | --- | --- | --- | --- | --- | --- | --- | --- | --- | --- | --- | --- | --- | --- |
|  | **DLS** | **INT** | **MLS** | **dVEN** | **vVEN** | **F-value** | ***P* value** | **DLS**  **vs.**  **INT** | **DLS**  **vs.**  **dVEN** | **DLS**  **vs.**  **vVEN** | **MLS**  **vs.**  **INT** | **MLS**  **vs.**  **dVEN** | **MLS**  **vs.**  **vVEN** | **DLS**  **vs.**  **MLS** | **INT**  **vs.**  **dVEN** | **INT**  **vs.**  **vVEN** | **dVEN**  **vs.**  **vVEN** |
| Fish 1 | 82.792 ± 16.166 | 18.427 ± 10.976 | 87.037 ± 32.190 | 10.978 ± 12.475 | 13.475 ± 5.048 | 66.61 | <0.001 | <0.001 | <0.001 | <0.001 | <0.001 | <0.001 | <0.001 | 0.886 | 0.899 | 0.996 | 1.000 |
| Fish2 | 69.706 ± 13.449 | 16.612 ± 10.870 | 77.800 ± 14.327 | 10.976 ± 8.095 | 0.000 ± 0.000 | 540.8 | <0.001 | <0.001 | <0.001 | <0.001 | <0.001 | <0.001 | <0.001 | <0.01 | 0.123 | <0.001 | <0.001 |
| Fish3 | 69.820 ± 14.592 | 28.740 ± 10.800 | 81.100 ± 14.658 | 23.100 ± 10.759 | 16.600 ± 8.918 | 274.1 | <0.001 | <0.001 | <0.001 | <0.001 | <0.001 | <0.001 | <0.001 | <0.001 | 0.150 | <0.001 | 0.095 |
| Fish4 | 70.480 ± 13.833 | 18.952 ± 10.087 | 79.320 ± 12.866 | 20.610 ± 10.793 | 12.097 ± 4.895 | 343.5 | <0.001 | <0.001 | <0.001 | <0.001 | <0.001 | <0.001 | <0.001 | <0.01 | 0.9639 | 0.085 | <0.05 |
| Fish5 | 65.200 ± 12.766 | 20.060 ± 8.730 | 70.560 ± 16.597 | 24.174 ± 11.949 | 13.289 ± 6.718 | 235.4 | <0.001 | <0.001 | <0.001 | <0.001 | <0.001 | <0.001 | <0.001 | 0.175 | 0.455 | 0.072 | <0.001 |
| Average | 71.600 ± 6.600 | 20.558 ± 4.740 | 79.163 ± 5.951 | 17.967 ± 6.511 | 11.092 ± 6.420 | 142.9 | <0.001 | <0.001 | <0.001 | <0.001 | <0.001 | <0.001 | <0.001 | 0.317 | 0.960 | 0.140 | 0.408 |

| dark morph | Melanophore dispersed diameter (µm) | | | | | ANOVA | | Tukey HSD *P* value | | | | | | | | | |
| --- | --- | --- | --- | --- | --- | --- | --- | --- | --- | --- | --- | --- | --- | --- | --- | --- | --- |
|  | **DLS** | **INT** | **MLS** | **dVEN** | **vVEN** | **F-value** | ***P* value** | **DLS**  **vs.**  **INT** | **DLS**  **vs.**  **dVEN** | **DLS**  **vs.**  **vVEN** | **MLS**  **vs.**  **INT** | **MLS**  **vs.**  **dVEN** | **MLS**  **vs.**  **vVEN** | **DLS**  **vs.**  **MLS** | **INT**  **vs.**  **dVEN** | **INT**  **vs.**  **vVEN** | **dVEN**  **vs.**  **vVEN** |
| Fish 1 | 59.390 ± 18.643 | 68.024 ± 11.557 | 63.532 ± 28.352 | 92.116 ± 24.425 | 113.502 ± 25.330 | 52.17 | <0.001 | 0.309 | <0.001 | <0.001 | 0.855 | <0.001 | <0.001 | 0.888 | <0.001 | <0.001 | <0.001 |
| Fish2 | 43.622 ± 19.783 | 84.736 ± 16.645 | 25.042 ± 16.534 | 101.048 ± 20.651 | 113.458 ± 24.214 | 183 | <0.001 | <0.001 | <0.001 | <0.001 | <0.001 | <0.001 | <0.001 | <0.001 | <0.001 | <0.001 | <0.05 |
| Fish3 | 48.120 ± 16.936 | 85.100 ± 15.036 | 34.840 ± 15.315 | 84.880 ± 17.842 | 89.160 ± 23.0415 | 98.6 | <0.001 | <0.001 | <0.001 | <0.001 | <0.001 | <0.001 | <0.001 | <0.01 | 1.000 | 0.787 | 0.753 |
| Fish4 | 45.780 ± 16.242 | 72.220 ± 13.239 | 24.940 ± 10.373 | 81.880 ± 14.728 | 89.040 ± 16.240 | 174.3 | <0.001 | <0.001 | <0.001 | <0.001 | <0.001 | <0.001 | <0.001 | <0.001 | <0.01 | <0.001 | 0.095 |
| Fish5 | 51.620 ± 14.051 | 80.100 ± 12.435 | 42.880 ± 16.090 | 88.900 ± 14.354 | 119.700 ± 24.576 | 166.4 | <0.001 | <0.001 | <0.001 | <0.001 | <0.001 | <0.001 | <0.001 | 0.075 | 0.072 | <0.001 | <0.001 |
| Average | 49.706 ± 6.172 | 78.036 ± 7.634 | 38.247 ± 15.999 | 89.765 ± 7.410 | 104.972 ± 14.710 | 30.82 | <0.001 | <0.01 | <0.001 | <0.001 | <0.001 | <0.001 | <0.001 | 0.501 | 0.479 | <0.01 | 0.238 |

| yellow morph | Xanthophore coverage (%) | | | | | ANOVA | | Tukey HSD *P* value | | | | | | | | | |
| --- | --- | --- | --- | --- | --- | --- | --- | --- | --- | --- | --- | --- | --- | --- | --- | --- | --- |
|  | **DLS** | **INT** | **MLS** | **dVEN** | **vVEN** | **F-value** | ***P* value** | **DLS**  **vs.**  **INT** | **DLS**  **vs.**  **dVEN** | **DLS**  **vs.**  **vVEN** | **MLS**  **vs.**  **INT** | **MLS**  **vs.**  **dVEN** | **MLS**  **vs.**  **vVEN** | **DLS**  **vs.**  **MLS** | **INT**  **vs.**  **dVEN** | **INT**  **vs.**  **vVEN** | **dVEN**  **vs.**  **vVEN** |
| Fish 1 | 0.062 ± 0.114 | 0.067 ± 0.085 | 0.306 ± 0.276 | 1.342 ± 2.133 | 7.012 ± 3.894 | 11.24 | <0.001 | 1.000 | 0.844 | <0.001 | 1.000 | 0.920 | <0.001 | 1.000 | 0.846 | <0.001 | <0.01 |
| Fish2 | 0.065 ± 0.093 | 0.170 ± 0.061 | 0.179 ± 0.079 | 3.362 ± 4.390 | 15.264 ± 6.362 | 17.97 | <0.001 | 1.000 | 0.569 | <0.001 | 1.000 | 0.601 | <0.001 | 1.000 | 0.599 | <0.001 | <0.001 |
| Fish3 | 0.361 ± 0.345 | 1.076 ± 0.897 | 0.063 ± 0.039 | 0.977 ± 0.653 | 10.780 ± 2.376 | 74.39 | <0.001 | 0.872 | 0.920 | <0.001 | 0.662 | 0.739 | <0.001 | 0.994 | 1.000 | <0.001 | <0.001 |
| Fish4 | 0.294 ± 0.189 | 2.713 ± 1.577 | 0.091 ± 0.091 | 0.734 ± 0.585 | 6.103 ± 3.098 | 12.78 | <0.001 | 0.150 | 0.992 | <0.001 | 0.103 | 0.966 | <0.001 | 1.000 | 0.311 | <0.05 | <0.001 |
| Fish5 | 0.480 ± 0.522 | 4.364 ± 3.748 | 0.674 ± 0.575 | 1.982 ± 0.887 | 16.943 ± 6.447 | 20.97 | <0.001 | 0.391 | 0.953 | <0.001 | 0.440 | 0.971 | <0.001 | 1.000 | 0.797 | <0.001 | <0.001 |
| Average | 0.252 ± 0.185 | 1.678 ± 1.838 | 0.262 ± 0.249 | 1.680 ± 1.051 | 11.220 ± 4.827 | 19.3 | <0.001 | 0.872 | 0.871 | <0.001 | 0.875 | 0.874 | <0.001 | 1.000 | 1.000 | <0.001 | <0.001 |

| dark morph | Xanthophore coverage (%) | | | | | ANOVA | | Tukey HSD *P* value | | | | | | | | | |
| --- | --- | --- | --- | --- | --- | --- | --- | --- | --- | --- | --- | --- | --- | --- | --- | --- | --- |
|  | **DLS** | **INT** | **MLS** | **dVEN** | **vVEN** | **F-value** | ***P* value** | **DLS**  **vs.**  **INT** | **DLS**  **vs.**  **dVEN** | **DLS**  **vs.**  **vVEN** | **MLS**  **vs.**  **INT** | **MLS**  **vs.**  **dVEN** | **MLS**  **vs.**  **vVEN** | **DLS**  **vs.**  **MLS** | **INT**  **vs.**  **dVEN** | **INT**  **vs.**  **vVEN** | **dVEN**  **vs.**  **vVEN** |
| Fish 1 | 0.345 ± 0.278 | 0.117 ± 0.078 | 0.189 ± 0.145 | 0.031 ± 0.022 | 0.049 ± 0.024 | 3.843 | <0.05 | 0.135 | <0.05 | <0.05 | 0.935 | 0.448 | 0.565 | 0.453 | 0.878 | 0.94 | 1.000 |
| Fish2 | 0.286 ± 0.252 | 0.060 ± 0.051 | 0.068 ± 0.019 | 0.122 ± 0.116 | 0.024 ± 0.017 | 3.322 | <0.05 | 0.072 | 0.283 | <0.05 | 1.000 | 0.959 | 0.981 | 0.086 | 0.936 | 0.991 | 0.738 |
| Fish3 | 0.284 ± 0.118 | 0.318 ± 0.1034 | 0.123 ± 0.216 | 0.055 ± 0.059 | 0.054 ± 0.037 | 5.247 | <0.05 | 0.99 | 0.056 | 0.054 | 0.130 | 0.9022 | 0.898 | 0.276 | <0.05 | <0.05 | 1.000 |
| Fish4 | 0.232 ± 0.346 | 0.086 ± 0.092 | 0.945 ± 1.602 | 0.373 ± 0.658 | 0.022 ± 0.014 | 1.087 | 0.39 | 0.998 | 0.998 | 0.993 | 0.446 | 0.782 | 0.377 | 0.619 | 0.977 | 1.000 | 0.954 |
| Fish5 | 5.698 ± 2.980 | 1.426 ± 0.809 | 2.439 ± 1.247 | 0.544 ± 0.339 | 0.544 ± 0.339 | 11.06 | <0.001 | <0.01 | <0.001 | <0.001 | 0.820 | 0.301 | 0.144 | <0.05 | 0.881 | 0.649 | 0.991 |
| Average | 1.369 ± 2.421 | 0.402 ± 0.582 | 0.757 ± 1.008 | 0.225 ± 0.224 | 0.054 ± 0.041 | 0.933 | 0.465 | 0.712 | 0.574 | 0.442 | 0.990 | 0.956 | 0.887 | 0.925 | 0.999 | 0.990 | 0.999­ |

| yellow morph | Xanthophore density (cells/mm^2^) | | | | | ANOVA | | Tukey HSD *P* value | | | | | | | | | |
| --- | --- | --- | --- | --- | --- | --- | --- | --- | --- | --- | --- | --- | --- | --- | --- | --- | --- |
|  | **DLS** | **INT** | **MLS** | **dVEN** | **vVEN** | **F-value** | ***P* value** | **DLS**  **vs.**  **INT** | **DLS**  **vs.**  **dVEN** | **DLS**  **vs.**  **vVEN** | **MLS**  **vs.**  **INT** | **MLS**  **vs.**  **dVEN** | **MLS**  **vs.**  **vVEN** | **DLS**  **vs.**  **MLS** | **INT**  **vs.**  **dVEN** | **INT**  **vs.**  **vVEN** | **dVEN**  **vs.**  **vVEN** |
| Fish 1 | 14.32 ± 5.10 | 15.90 ± 7.77 | 10.39 ± 5.32 | 9.87 ± 11.42 | 55.74 ± 20.75 | 14 | <0.001 | 1.000 | 0.973 | <0.001 | 0.942 | 1.000 | <0.001 | 0.983 | 0.922 | <0.001 | <0.001 |
| Fish2 | 23.58 ± 7.27 | 41.01 ± 9.25 | 8.68 ± 5.32 | 21.07 ± 16.05 | 144.11 ± 48.89 | 26.99 | <0.001 | 0.772 | 1.000 | <0.001 | 0.237 | 0.920 | <0.001 | 0.855 | 0.677 | <0.001 | <0.001 |
| Fish3 | 37.57 ± 32.51 | 118.65 ± 32.60 | 9.91 ± 3.78 | 37.97 ± 29.20 | 97.52 ± 15.89 | 16.1 | <0.001 | <0.001 | 1.000 | <0.05 | <0.001 | 0.432 | <0.001 | 0.446 | <0.001 | 0.687 | <0.05 |
| Fish4 | 40.01 ± 27.45 | 90.62 ± 26.66 | 17.18 ± 6.04 | 19.47 ± 5.69 | 81.36 ± 16.61 | 16.39 | <0.001 | <0.01 | 0.452 | <0.05 | <0.001 | 1.000 | <0.001 | 0.350 | <0.001 | 0.936 | <0.001 |
| Fish5 | 37.33 ± 23.75 | 133.14 ± 20.82 | 40.22 ± 36.03 | 96.07 ± 55.99 | 117.72 ± 19.86 | 8.356 | <0.001 | <0.01 | 0.086 | <0.05 | <0.01 | 0.111 | <0.05 | 1.000 | 0.47 | 0.951 | 0.851 |
| Average | 30.56 ± 11.14 | 79.86 ± 50.15 | 17.28 ± 13.25 | 36.89 ± 34.60 | 99.29 ± 33.80 | 5.946 | <0.01 | 0.149 | 0.998 | <0.05 | <0.05 | 0.857 | <0.01 | 0.964 | 0.252 | 0.871 | <0.05 |

| dark morph | Xanthophore density (cells/mm^2^) | | | | | ANOVA | | Tukey HSD *P* value | | | | | | | | | |
| --- | --- | --- | --- | --- | --- | --- | --- | --- | --- | --- | --- | --- | --- | --- | --- | --- | --- |
|  | **DLS** | **INT** | **MLS** | **dVEN** | **vVEN** | **F-value** | ***P* value** | **DLS**  **vs.**  **INT** | **DLS**  **vs.**  **dVEN** | **DLS**  **vs.**  **vVEN** | **MLS**  **vs.**  **INT** | **MLS**  **vs.**  **dVEN** | **MLS**  **vs.**  **vVEN** | **DLS**  **vs.**  **MLS** | **INT**  **vs.**  **dVEN** | **INT**  **vs.**  **vVEN** | **dVEN**  **vs.**  **vVEN** |
| Fish 1 | 15.29 ± 9.85 | 19.02 ± 20.50 | 5.93 ± 3.75 | 5.69 ± 4.49 | 2.85 ± 0.80 | 2.211 | 0.104 | 0.979 | 0.608 | 0.363 | 0.315 | 1.000 | 0.990 | 0.630 | 0.299 | 0.147 | 0.992 |
| Fish2 | 14.16 ± 13.52 | 3.68 ± 1.84 | 6.25 ± 9.09 | 2.97 ± 1.09 | 1.13 ± 0.54 | 2.411 | 0.083 | 0.201 | 0.155 | 0.073 | 0.980 | 0.953 | 0.803 | 0.456 | 1.000 | 0.981 | 0.994 |
| Fish3 | 29.22 ± 15.34 | 17.37 ± 9.45 | 5.61 ± 1.75 | 6.06 ± 3.00 | 4.14 ± 1.20 | 8.544 | <0.001 | 0.135 | <0.01 | <0.001 | 0.199 | 1.000 | 0.998 | <0.01 | 0.229 | 0.120 | 0.996 |
| Fish4 | 26.45 ± 11.90 | 6.65 ± 4.50 | 23.14 ± 29.92 | 9.30 ± 9.95 | 1.47 ± 1.26 | 2.538 | 0.072 | 0.276 | 0.411 | 0.109 | 0.448 | 0.612 | 0.202 | 0.997 | 0.999 | 0.982 | 0.923 |
| Fish5 | 193.73 ± 72.68 | 92.32 ± 14.49 | 118.91 ± 11.63 | 58.42 ± 20.57 | 2.33 ± 1.30 | 20.9 | <0.001 | <0.01 | <0.001 | <0.001 | 0.746 | 0.081 | <0.001 | <0.05 | 0.550 | <0.01 | 0.119 |
| Average | 55.77 ± 7.41 | 27.81 ± 36.67 | 31.97 ± 49.17 | 16.49 ± 23.55 | 2.38 ± 1.20 | 0.951 | 0.455 | 0.864 | 0.654 | 0.370 | 1.000 | 0.982 | 0.838 | 0.919 | 0.994 | 0.899 | 0.987 |

| yellow morph | Xanthophore dispersed diameter (µm) | | | | | ANOVA | | Tukey HSD *P* value | | | | | | | | | |
| --- | --- | --- | --- | --- | --- | --- | --- | --- | --- | --- | --- | --- | --- | --- | --- | --- | --- |
|  | **DLS** | **INT** | **MLS** | **dVEN** | **vVEN** | **F-value** | ***P* value** | **DLS**  **vs.**  **INT** | **DLS**  **vs.**  **dVEN** | **DLS**  **vs.**  **vVEN** | **MLS**  **vs.**  **INT** | **MLS**  **vs.**  **dVEN** | **MLS**  **vs.**  **vVEN** | **DLS**  **vs.**  **MLS** | **INT**  **vs.**  **dVEN** | **INT**  **vs.**  **vVEN** | **dVEN**  **vs.**  **vVEN** |
| Fish 1 | 13.300 ± 10.077 | 9.350 ± 6.542 | 21.694 ± 17.209 | 34.777 ± 36.327 | 80.962 ± 30.878 | 42.96 | <0.001 | 0.992 | 0.055 | <0.001 | 0.472 | 0.250 | <0.001 | 0.843 | <0.01 | <0.001 | <0.001 |
| Fish2 | 9.770 ± 7.013 | 7.922 ± 3.992 | 20.500 ± 8.252 | 30.745 ± 19.451 | 71.040 ± 27.801 | 124.3 | <0.001 | 0.980 | <0.001 | <0.001 | <0.01 | <0.05 | <0.001 | <0.05 | <0.001 | <0.001 | <0.001 |
| Fish3 | 12.870 ± 7.060 | 21.680 ± 6.796 | 21.318 ± 11.586 | 28.231 ± 10.207 | 63.100 ± 16.032 | 27.8 | <0.001 | <0.05 | <0.001 | <0.001 | 1.000 | 0.150 | <0.001 | 0.092 | 0.055 | <0.001 | <0.001 |
| Fish4 | 28.103 ± 9.363 | 32.380 ± 7.887 | 21.750 ± 8.848 | 39.686 ± 16.055 | 45.440 ± 16.371 | 20.25 | <0.001 | 0.497 | <0.001 | <0.001 | <0.01 | <0.001 | <0.001 | 0.289 | 0.065 | <0.001 | 0.228 |
| Fish5 | 19.658 ± 5.293 | 27.460 ± 8.227 | 20.156 ± 8.345 | 32.720 ± 21.051 | 62.490 ± 16.424 | 78.65 | <0.001 | 0.063 | <0.001 | <0.001 | 0.072 | <0.001 | <0.001 | 1.000 | 0.304 | <0.001 | <0.001 |
| Average | 16.740 ± 7.298 | 19.758 ± 10.848 | 21.083 ± 0.720 | 33.232 ± 4.344 | 64.606 ± 13.067 | 27.04 | <0.001 | 0.979 | <0.05 | <0.001 | 0.999 | 0.199 | <0.001 | 0.925 | 0.129 | <0.001 | <0.001 |

| dark morph | Xanthophore dispersed diameter (µm) | | | | | ANOVA | | Tukey HSD *P* value | | | | | | | | | |
| --- | --- | --- | --- | --- | --- | --- | --- | --- | --- | --- | --- | --- | --- | --- | --- | --- | --- |
|  | **DLS** | **INT** | **MLS** | **dVEN** | **vVEN** | **F-value** | ***P* value** | **DLS**  **vs.**  **INT** | **DLS**  **vs.**  **dVEN** | **DLS**  **vs.**  **vVEN** | **MLS**  **vs.**  **INT** | **MLS**  **vs.**  **dVEN** | **MLS**  **vs.**  **vVEN** | **DLS**  **vs.**  **MLS** | **INT**  **vs.**  **dVEN** | **INT**  **vs.**  **vVEN** | **dVEN**  **vs.**  **vVEN** |
| Fish 1 | 6.000 ± 4.872 | 9.941 ± 6.206 | 9.283 ± 4.773 | 7.127 ± 2.691 | 15.662 ± 7.292 | 11.08 | <0.001 | 0.079 | 0.946 | <0.001 | 0.987 | 0.460 | <0.001 | 0.170 | 0.243 | <0.01 | <0.001 |
| Fish2 | 16.706 ± 7.991 | 12.712 ± 4.765 | 22.193 ± 7.385 | 17.505 ± 6.231 | 16.167 ± 4.703 | 5.842 | <0.001 | 0.262 | 0.993 | 0.998 | <0.001 | 0.129 | <0.05 | <0.05 | 0.197 | 0.510 | 0.968 |
| Fish3 | 14.897 ± 5.871 | 14.047 ± 6.028 | 18.029 ± 7.163 | 16.387 ± 6.601 | 15.780 ± 9.410 | 1.614 | 0.172 | 0.984 | 0.914 | 0.980 | 0.124 | 0.893 | 0.635 | 0.357 | 0.651 | 0.782 | 0.996 |
| Fish4 | 12.844 ± 6.306 | 8.162 ± 4.330 | 21.220 ± 7.505 | 16.278 ± 7.682 | 16.422 ± 5.883 | 22.11 | <0.001 | <0.05 | 0.124 | 0.068 | <0.001 | <0.01 | <0.01 | <0.001 | <0.001 | <0.001 | 1.000 |
| Fish5 | 28.820 ± 10.617 | 20.660 ± 7.883 | 31.500 ± 14.380 | 20.820 ± 8.113 | 26.867 ± 17.828 | 8.433 | <0.001 | <0.05 | <0.01 | 0.952 | <0.001 | <0.001 | 0.433 | 0.786 | 1.000 | 0.154 | 0.174 |
| Average | 15.854 ± 8.305 | 13.104 ± 4.810 | 20.445 ± 8.004 | 15.623 ± 5.093 | 18.179 ± 4.866 | 0.941 | 0.46 | 0.959 | 1.000 | 0.978 | 0.396 | 0.758 | 0.980 | 0.788 | 0.970 | 0.723 | 0.968 |

| yellow morph | Iridophore coverage (%) | | | | | ANOVA | | Tukey HSD *P* value | | | | | | | | | |
| --- | --- | --- | --- | --- | --- | --- | --- | --- | --- | --- | --- | --- | --- | --- | --- | --- | --- |
|  | **DLS** | **INT** | **MLS** | **dVEN** | **vVEN** | **F-value** | ***P* value** | **DLS**  **vs.**  **INT** | **DLS**  **vs.**  **dVEN** | **DLS**  **vs.**  **vVEN** | **MLS**  **vs.**  **INT** | **MLS**  **vs.**  **dVEN** | **MLS**  **vs.**  **vVEN** | **DLS**  **vs.**  **MLS** | **INT**  **vs.**  **dVEN** | **INT**  **vs.**  **vVEN** | **dVEN**  **vs.**  **vVEN** |
| Fish 1 | 0.129 ± 0.234 | 0.465 ± 0.639 | 0.002 ± 0.003 | 0.022 ± 0.031 | 0.093 ± 0.047 | 1.889 | 0.152 | 0.433 | 0.981 | 1.000 | 0.157 | 1.000 | 0.989 | 0.963 | 0.188 | 0.337 | 0.996 |
| Fish2 | 0.510 ± 0.558 | 3.562 ± 1.900 | 0.066 ± 0.129 | 0.316 ± 0.334 | 0.102 ± 0.084 | 13.73 | <0.001 | <0.001 | 0.997 | 0.950 | <0.001 | 0.992 | 1.000 | 0.934 | <0.001 | <0.001 | 0.995 |
| Fish3 | 0.032 ± 0.031 | 0.637 ± 0.652 | 0.001 ± 0.002 | 0.038 ± 0.070 | 0.263 ± 0.311 | 3.405 | <0.05 | 0.057 | 1.000 | 0.798 | <0.05 | 1.000 | 0.710 | 1.000 | 0.059 | 0.391 | 0.808 |
| Fish4 | 0.030 ± 0.056 | 0.598 ± 0.504 | 0.01 ± 0.015 | 0.180 ± 0.203 | 0.129 ± 0.149 | 4.437 | <0.01 | <0.05 | 0.878 | 0.970 | <0.05 | 0.828 | 0.9463 | 1.000 | 0.107 | 0.057 | 0.998 |
| Fish5 | 0.050 ± 0.068 | 0.419 ± 0.619 | 0.014 ± 0.031 | 0.043 ± 0.040 | 0.037 ± 0.043 | 1.878 | 0.154 | 0.265 | 1.000 | 1.000 | 0.192 | 1.000 | 1.000 | 1.000 | 0.250 | 0.236 | 1.000 |
| Average | 0.150 ± 0.205 | 1.136 ± 1.359 | 0.019 ± 0.027 | 0.120 ± 0.127 | 0.125 ± 0.084 | 2.819 | 0.0527 | 0.126 | 1.000 | 1.000 | 0.066 | 0.999 | 0.999 | 0.997 | 0.109 | 0.111 | 1.000 |

| dark morph | Iridophore coverage (%) | | | | | ANOVA | | Tukey HSD *P* value | | | | | | | | | |
| --- | --- | --- | --- | --- | --- | --- | --- | --- | --- | --- | --- | --- | --- | --- | --- | --- | --- |
|  | **DLS** | **INT** | **MLS** | **dVEN** | **vVEN** | **F-value** | ***P* value** | **DLS**  **vs.**  **INT** | **DLS**  **vs.**  **dVEN** | **DLS**  **vs.**  **vVEN** | **MLS**  **vs.**  **INT** | **MLS**  **vs.**  **dVEN** | **MLS**  **vs.**  **vVEN** | **DLS**  **vs.**  **MLS** | **INT**  **vs.**  **dVEN** | **INT**  **vs.**  **vVEN** | **dVEN**  **vs.**  **vVEN** |
| Fish 1 | 2.718 ± 1.177 | 1.655 ± 0.893 | 0.692 ± 0.621 | 0.128 ± 0.150 | 0.496 ± 0.181 | 10.46 | <0.001 | 0.179 | <0.001 | <0.001 | 0.259 | 0.734 | 0.992 | <0.01 | <0.05 | 0.124 | 0.927 |
| Fish2 | 4.098 ± 3.759 | 0.271 ± 0.179 | 0.359 ± 0.344 | 0.103 ± 0.206 | 0.018 ± 0.021 | 5.368 | <0.01 | <0.05 | <0.05 | <0.01 | 1.000 | 0.999 | 0.998 | <0.05 | 1.000 | 0.999 | 1.000 |
| Fish3 | 2.295 ± 1.795 | 0.684 ± 0.724 | 0.275 ± 0.164 | 0.122 ± 0.170 | 0.068 ± 0.045 | 5.679 | <0.01 | 0.058 | <0.01 | <0.01 | 0.944 | 0.999 | 0.995 | <0.05 | 0.844 | 0.796 | 1.000 |
| Fish4 | 5.049 ± 1.059 | 0.631 ± 0.548 | 1.75 ± 1.95 | 0.286 ± 0.319 | 0.149 ± 0.102 | 19.55 | <0.001 | <0.001 | <0.001 | <0.001 | 0.448 | 0.205 | 0.142 | <0.001 | 0.984 | 0.945 | 1.000 |
| Fish5 | 2.663 ± 0.892 | 0.764 ± 0.543 | 0.708 ± 0.397 | 0.108 ± 0.127 | 0.022 ± 0.033 | 22.5 | <0.001 | <0.001 | <0.001 | <0.001 | 1.000 | 0.357 | 0.237 | <0.001 | 0.275 | 0.176 | 0.999 |
| Average | 3.365 ± 1.165 | 0.801 ± 0.513 | 0.757 ± 0.588 | 0.150 ± 0.077 | 0.151 ± 0.200 | 22.12 | <0.001 | <0.001 | <0.001 | <0.001 | 1.000 | 0.566 | 0.568 | <0.001 | 0.501 | 0.502 | 1.000 |

**Supplementary Table S2. Statistical tests of chromatophore measurement (across morph)**

| **Measurement** | **position** | **Mean value ± SD** | | ***P* value**  **(two-tailed *t* test)** | **dark/yellow**  **Fold change** |
| --- | --- | --- | --- | --- | --- |
|  |  | **dark morph** | **yellow morph** |  |  |
| **Melanophore coverage**  **(%)** | DLS | 29.397 ± 7.926 | 84.690 ± 6.761 | <0.001 | 0.347 |
|  | INT | 73.239 ± 8.519 | 1.343 ± 1.455 | <0.001 | 54.534 |
|  | MLS | 10.653 ± 6.612 | 79.313 ± 9.873 | <0.001 | 0.1343 |
|  | dVEN | 69.356 ± 9.122 | 0.607 ± 0.493 | <0.001 | 114.260 |
|  | vVEN | 69.883 ± 5.016 | 0.215 ± 0.153 | <0.001 | 325.037 |
| **Melanophore density**  **(cell/mm^2^)** | DLS | 186.36 ± 43.02 | 377.34 ± 50.27 | <0.001 | 0.494 |
|  | INT | 301.71 ± 60.14 | 52.83 ± 39.78 | <0.001 | 5.711 |
|  | MLS | 81.64 ± 51.09 | 287.67 ± 51.11 | <0.001 | 0.284 |
|  | dVEN | 212.81± 27.49 | 22.93 ± 13.71 | <0.001 | 9.281 |
|  | vVEN | 134.70 ± 13.01 | 3.49 ± 2.88 | <0.001 | 38.596 |
| **Melanophore dispersed diameter**  **(µm)** | DLS | 49.706 ± 6.172 | 71.600 ± 6.600 | <0.001 | 0.694 |
|  | INT | 78.036 ± 7.634 | 20.558 ± 4.740 | <0.001 | 3.796 |
|  | MLS | 38.247 ± 15.999 | 79.163 ± 5.951 | <0.01 | 0.483 |
|  | dVEN | 89.765 ± 7.410 | 17.967 ± 6.511 | <0.001 | 4.996 |
|  | vVEN | 104.972 ± 14.710 | 11.092 ± 6.420 | <0.001 | 9.464 |
| **Xanthophore coverage**  **(%)** | DLS | 1.369 ± 2.421 | 0.252 ± 0.185 | 0.361 | 5.433 |
|  | INT | 0.402 ± 0.582 | 1.678 ± 1.838 | 0.201 | 0.240 |
|  | MLS | 0.757 ± 1.008 | 0.262 ± 0.249 | 0.345 | 2.889 |
|  | dVEN | 0.225 ± 0.224 | 1.680 ± 1.051 | <0.05 | 0.134 |
|  | vVEN | 0.054 ± 0.041 | 11.220 ± 4.827 | <0.01 | 0.005 |
| **Xanthophore density**  **(cell/mm^2^)** | DLS | 55.77 ± 7.41 | 30.56 ± 11.14 | 0.510 | 1.825 |
|  | INT | 27.81 ± 36.67 | 79.86 ± 50.15 | 0.102 | 0.348 |
|  | MLS | 31.97 ± 49.17 | 17.28 ± 13.25 | 0.550 | 1.850 |
|  | dVEN | 16.49 ± 23.55 | 36.89 ± 34.60 | 0.312 | 0.447 |
|  | vVEN | 2.38 ± 1.20 | 99.29 ± 33.80 | <0.01 | 0.024 |
| **Xanthophore dispersed diameter**  **(µm)** | DLS | 15.854 ± 8.305 | 16.740 ± 7.298 | 0.862 | 0.947 |
|  | INT | 13.104 ± 4.810 | 19.758 ± 10.848 | 0.261 | 0.663 |
|  | MLS | 20.445 ± 8.004 | 21.083 ± 0.720 | 0.868 | 0.970 |
|  | dVEN | 15.623 ± 5.093 | 33.232 ± 4.344 | <0.001 | 0.470 |
|  | vVEN | 18.179 ± 4.866 | 64.606 ± 13.067 | <0.001 | 0.281 |
| **Iridophore coverage**  **(%)** | DLS | 3.365 ± 1.165 | 0.150 ± 0.205 | <0.01 | 22.433 |
|  | INT | 0.801 ± 0.513 | 1.136 ± 1.359 | 0.627 | 0.7051 |
|  | MLS | 0.757 ± 0.588 | 0.019 ± 0.027 | <0.05 | 39.842 |
|  | dVEN | 0.150 ± 0.077 | 0.120 ± 0.127 | 0.667 | 1.250 |
|  | vVEN | 0.151 ± 0.200 | 0.125 ± 0.084 | 0.800 | 1.208 |

**Supplementary Table S3. Axon density and results of the statistical tests (within morph/sex and individual)**

| ***M. auratus* (WT)**  **yellow morph** | **AcTub^+^ fibers / cm (Anterior)** | | | | **ANOVA** | | **Tukey HSD *P* value** | | |
| --- | --- | --- | --- | --- | --- | --- | --- | --- | --- |
|  | **Dorsum** | **Medium** | **Ventrum** | **Whole trunk** | **F-value** | ***P* value** | **D vs. M** | **D vs. V** | **M vs. V** |
| **Fish 1** | 115.08 ± 29.90 | 99.48 ± 13.84 | 127.63 ± 29.23 | 114.07 ± 26.39 | 1.537 | 0.255 | 0.609 | 0.722 | 0.228 |
| **Fish 2** | 75.72 ± 6.82 | 91.91 ± 13.79 | 127.94 ± 28.36 | 98.52 ± 28.42 | 10.3 | <0.01 | 0.384 | <0.01 | <0.05 |
| **Fish 3** | 84.94 ± 24.07 | 93.20 ± 13.13 | 96.07 ± 21.75 | 91.51 ± 18.99 | 0.432 | 0.658 | 0.773 | 0.655 | 0.969 |
| **Fish 4** | 85.40 ± 13.02 | 87.47 ± 12.63 | 119.90 ± 21.11 | 97.59 ± 22.11 | 7.246 | <0.01 | 0.977 | <0.05 | <0.05 |
| **Fish 5** | 64.31 ± 26.94 | 74.44 ± 19.27 | 95.10 ± 6.60 | 77.95 ± 22.40 | 3.239 | 0.075 | 0.698 | 0.067 | 0.254 |
| **Average** | 85.09 ± 18.84 | 89.30 ± 9.35 | 113.33 ± 16.52 | 95.93 ± 13.05 | 4.867 | <0.05 | 0.903 | <0.05 | 0.072 |

| ***M. auratus* (WT)**  **dark morph** | **AcTub^+^ fibers / cm (Anterior)** | | | | **ANOVA** | | **Tukey HSD *P* value** | | |
| --- | --- | --- | --- | --- | --- | --- | --- | --- | --- |
|  | **Dorsum** | **Medium** | **Ventrum** | **Whole trunk** | **F-value** | ***P* value** | **D vs. M** | **D vs. V** | **M vs. V** |
| **Fish 1** | 129.80 ± 22.98 | 147.97 ± 27.90 | 164.29 ± 17.44 | 147.35 ± 25.94 | 2.772 | 0.102 | 0.453 | 0.86 | 0.524 |
| **Fish 2** | 146.23 ± 16.30 | 131.74 ± 13.47 | 150.55 ± 25.19 | 142.29 ± 18.81 | 1.352 | 0.299 | 0.450 | 0.934 | 0.315 |
| **Fish 3** | 156.98 ± 11.92 | 150.52 ± 20.30 | 164.90 ± 12.57 | 157.47 ± 15.51 | 1.092 | 0.367 | 0.789 | 0.703 | 0.336 |
| **Fish 4** | 139.67 ± 19.26 | 142.87 ± 17.96 | 142.31 ± 29.82 | 141.70 ± 21.11 | 0.03 | 0.971 | 0.970 | 0.981 | 0.999 |
| **Fish 5** | 144.86 ± 20.29 | 129.78 ± 9.41 | 144.53 ± 15.58 | 139.71 ± 16.15 | 1.876 | 0.187 | 0.268 | 0.999 | 0.229 |
| **Average** | 143.51 ± 9.92 | 124.38 ± 16.45 | 153.32 ± 10.73 | 145.70 ± 7.16 | 2.212 | 0.152 | 0.890 | 0.305 | 0.153 |

| ***M. auratus* (AM)**  **‘yellow morph’** | **AcTub^+^ fibers / cm (Anterior)** | | | | **ANOVA** | | **Tukey HSD *P* value** | | |
| --- | --- | --- | --- | --- | --- | --- | --- | --- | --- |
|  | **Dorsum** | **Medium** | **Ventrum** | **Whole trunk** | **F-value** | ***P* value** | **D vs. M** | **D vs. V** | **M vs. V** |
| **Fish 1** | 95.73 ± 21.43 | 111.54 ± 7.44 | 88.44 ± 25.76 | 98.57 ± 20.89 | 1.775 | 0.211 | 0.442 | 0.832 | 0.179 |
| **Fish 2** | 86.30 ± 44.06 | 82.70 ± 17.67 | 101.76 ± 20.63 | 90.25 ± 28.96 | 0.574 | 0.578 | 0.980 | 0.700 | 0.586 |
| **Fish 3** | 110.56 ± 8.29 | 91.94 ± 12.92 | 97.49 ± 20.32 | 99.99 ± 15.83 | 2.113 | 0.164 | 0.154 | 0.369 | 0.824 |
| **Fish 4** | 97.87 ± 15.31 | 3.82 ± 5.00 | 87.50 ± 17.15 | 93.06 ± 13.33 | 0.739 | 0.498 | 0.886 | 0.472 | 0.748 |
| **Average** | 97.61 ± 9.98 | 89.30 ± 9.35 | 93.80 ± 6.96 | 95.47 ± 4.58 | 0.156 | 0.858 | 0.926 | 0.851 | 0.984 |

| ***M. auratus* (AM)**  **‘dark morph’** | **AcTub^+^ fibers / cm (Anterior)** | | | | **ANOVA** | | **Tukey HSD *P* value** | | |
| --- | --- | --- | --- | --- | --- | --- | --- | --- | --- |
|  | **Dorsum** | **Medium** | **Ventrum** | **Whole trunk** | **F-value** | ***P* value** | **D vs. M** | **D vs. V** | **M vs. V** |
| **Fish 1** | 123.47 ± 11.81 | 132.90 ± 24.96 | 126.01 ± 15.65 | 127.21 ± 17.17 | 0.394 | 0.682 | 0.668 | 0.970 | 0.818 |
| **Fish 2** | 127.18 ± 23.39 | 133.05 ± 10.47 | 135.02 ± 15.11 | 131.83 ± 15.95 | 0.299 | 0.747 | 0.834 | 0.745 | 0.979 |
| **Fish 3** | 127.88 ± 19.39 | 116.92 ± 20.43 | 145.83 ± 8.60 | 130.21 ± 20.00 | 3.683 | 0.057 | 0.579 | 0.256 | <0.05 |
| **Fish 4** | 141.90 ± 18.83 | 154.06 ± 17.14 | 127.84 ± 20.00 | 141.27 ± 20.56 | 2.462 | 0.127 | 0.574 | 0.482 | 0.108 |
| **Fish 5** | 137.72 ± 19.50 | 119.06 ± 6.13 | 140.69 ± 13.80 | 132.49 ± 16.49 | 3.388 | 0.068 | 0.138 | 0.942 | 0.079 |
| **Average** | 131.63 ± 7.79 | 131.20 ± 14.83 | 135.08 ± 8.39 | 132.60 ± 5.26 | 0.193 | 0.827 | 0.998 | 0.871 | 0.840 |

| ***M. cyaneorhabdos***  **Female** | **AcTub^+^ fibers / cm (Anterior)** | | | | **ANOVA** | | **Tukey HSD *P* value** | | |
| --- | --- | --- | --- | --- | --- | --- | --- | --- | --- |
|  | **Dorsum** | **Medium** | **Ventrum** | **Whole trunk** | **F-value** | ***P* value** | **D vs. M** | **D vs. V** | **M vs. V** |
| Fish 1 | 128.64 ± 22.07 | 118.43 ± 23.58 | 121.49 ± 20.40 | 122.77 ± 20.86 | 0.287 | 0.755 | 0.747 | 0.854 | 0.971 |
| Fish 2 | 127.91 ± 18.59 | 118.49 ± 14.80 | 121.61 ± 31.63 | 122.67 ± 21.53 | 0.22 | 0.805 | 0.795 | 0.901 | 0.975 |
| Fish 3 | 156.80 ± 18.62 | 125.58 ± 27.00 | 102.45 ± 35.62 | 128.28 ± 34.66 | 4.76 | <0.05 | 0.222 | <0.05 | 0.417 |
| Fish 4 | 145.05 ± 41.57 | 91.91 ± 28.15 | 132.54 ± 20.67 | 123.17 ± 37.33 | 3.926 | <0.05 | <0.05 | 0.806 | 0.143 |
| Fish 5 | 116.54 ± 18.19 | 103.29 ± 27.83 | 90.50 ± 22.87 | 103.44 ± 24.21 | 1.562 | 0.25 | 0.651 | 0.221 | 0.670 |
| Average | 134.99 ± 15.87 | 111.5413.66 | 113.72 ± 16.90 | 120.07 ± 9.59 | 3.475 | 0.065 | 0.082 | 0.118 | 0.973 |

| ***M. cyaneorhabdos***  **Male** | **AcTub^+^ fibers / cm (Anterior)** | | | | **ANOVA** | | **Tukey HSD *P* value** | | |
| --- | --- | --- | --- | --- | --- | --- | --- | --- | --- |
|  | **Dorsum** | **Medium** | **Ventrum** | **Whole trunk** | **F-value** | ***P* value** | **D vs. M** | **D vs. V** | **M vs. V** |
| Fish 1 | 110.36 ± 23.87 | 111.61 ± 17.61 | 120.46 ± 19.68 | 113.91 ± 19.92 | 0.364 | 0.701 | 0.995 | 0.709 | 0.784 |
| Fish 2 | 134.97 ± 19.64 | 120.18 ± 21.29 | 117.68 ± 16.51 | 124.94 ± 19.68 | 1.321 | 0.3 | 0.437 | 0.331 | 0.977 |
| Fish 3 | 146.20 ± 16.67 | 122.06 ± 10.98 | 72.29 ± 61.87 | 113.52 ± 47.14 | 5.042 | <0.05 | 0.581 | <0.05 | 0.132 |
| Fish 4 | 123.17 ± 15.98 | 104.65 ± 15.68 | 106.70 ± 25.69 | 111.21 ± 20.59 | 1.29 | 0.308 | 0.346 | 0.396 | 0.985 |
| Fish 5 | 107.19 ± 49.96 | 123.02 ± 10.30 | 107.41 ± 23.18 | 112.21 ± 32.56 | 0.367 | 0.699 | 0.728 | 1.000 | 0.753 |
| Average | 124.38 ± 16.45 | 116.30 ± 7.92 | 104.91 ± 19.22 | 115.16 ± 5.58 | 2.043 | 0.172 | 0.690 | 0.152 | 0.488 |

| ***M. auratus* (WT)**  **yellow morph** | **AcTub^+^ fibers / cm (Posterior)** | | | | **ANOVA** | | **Tukey HSD *P* value** | | |
| --- | --- | --- | --- | --- | --- | --- | --- | --- | --- |
|  | **Dorsum** | **Medium** | **Ventrum** | **Whole trunk** | **F-value** | ***P* value** | **D vs. M** | **D vs. V** | **M vs. V** |
| **Fish 1** | 96.74 ± 32.79 | 77.50 ± 15.08 | 76.84 ± 25.57 | 83.69 ± 25.50 | 0.979 | 0.404 | 0.481 | 0.458 | 0.999 |
| **Fish 2** | 79.77 ± 21.72 | 72.44 ± 14.74 | 99.39 ± 21.92 | 83.87 ± 21.75 | 2.491 | 0.125 | 0.829 | 0.295 | 0.120 |
| **Fish 3** | 86.02 ± 15.81 | 56.07 ± 16.20 | 70.23 ± 18.27 | 69.85 ± 20.16 | 4.362 | <0.05 | <0.05 | 0.327 | 0.371 |
| **Fish 4** | 114.22 ± 18.47 | 76.13 ± 18.26 | 86.27 ± 22.04 | 92.21 ± 24.69 | 5.029 | <0.05 | <0.05 | 0.103 | 0.702 |
| **Fish 5** | 67.63 ± 20.66 | 68.44 ± 22.02 | 63.82 ± 20.88 | 66.63 ± 19.73 | 0.068 | 0.935 | 0.998 | 0.957 | 0.937 |
| **Average** | 88.88 ± 17.65 | 70.12 ± 8.61 | 79.31 ± 13.96 | 79.25 ± 10.68 | 2.273 | 0.146 | 0.125 | 0.540 | 0.564 |

| ***M. auratus* (WT)**  **dark morph** | **AcTub^+^ fibers / cm (Posterior)** | | | | **ANOVA** | | **Tukey HSD *P* value** | | |
| --- | --- | --- | --- | --- | --- | --- | --- | --- | --- |
|  | **Dorsum** | **Medium** | **Ventrum** | **Whole trunk** | **F-value** | ***P* value** | **D vs. M** | **D vs. V** | **M vs. V** |
| **Fish 1** | 143.34 ± 26.30 | 133.05 ± 15.88 | 143.72 ± 36.47 | 140.04 ± 26.00 | 0.242 | 0.789 | 0.828 | 1.000 | 0.816 |
| **Fish 2** | 166.97 ± 45.70 | 136.54 ± 9.79 | 133.47 ± 18.18 | 146.53 ± 31.62 | 1.848 | 0.203 | 0.279 | 0.256 | 0.987 |
| **Fish 3** | 145.82 ± 15.64 | 149.91 ± 27.30 | 138.91 ± 11.05 | 144.88 ± 18.43 | 0.147 | 0.668 | 0.940 | 0.840 | 0.648 |
| **Fish 4** | 158.82 ± 30.00 | 129.21 ± 16.60 | 144.67 ± 26.00 | 143.29 ± 25.93 | 2.036 | 0.17 | 0.149 | 0.638 | 0.560 |
| **Fish 5** | 159.71 ± 31.21 | 132.63 ± 29.87 | 156.75 ± 17.58 | 149.53 ± 27.45 | 1.907 | 0.183 | 0.232 | 0.979 | 0.252 |
| **Average** | 154.93 ± 10.00 | 136.27 ± 8.06 | 143.50 ± 8.64 | 144.85 ± 3.55 | 5.543 | <0.05 | <0.05 | 0.149 | 0.432 |

| ***M. auratus* (AM)**  **‘yellow morph’** | **AcTub^+^ fibers / cm (Posterior)** | | | | **ANOVA** | | **Tukey HSD *P* value** | | |
| --- | --- | --- | --- | --- | --- | --- | --- | --- | --- |
|  | **Dorsum** | **Medium** | **Ventrum** | **Whole trunk** | **F-value** | ***P* value** | **D vs. M** | **D vs. V** | **M vs. V** |
| **Fish 1** | 94.72 ± 37.87 | 93.03 ± 18.26 | 78.23 ± 22.22 | 88.66 ± 26.55 | 0.546 | 0.593 | 0.995 | 0.621 | 0.679 |
| **Fish 2** | 105.51 ± 45.97 | 58.37 ± 15.84 | 94.58 ± 27.26 | 86.15 ± 36.37 | 2.938 | 0.092 | 0.092 | 0.855 | 0.218 |
| **Fish 3** | 95.44 ± 23.30 | 87.28 ± 17.16 | 66.99 ± 29.63 | 83.23 ± 25.37 | 1.876 | 0.195 | 0.854 | 0.186 | 0.400 |
| **Fish 4** | 82.66 ± 14.02 | 69.40 ± 0.38 | 63.22 ± 20.86 | 71.76 ± 19.22 | 1.413 | 0.281 | 0.519 | 0.265 | 0.862 |
| **Average** | 94.58 ± 9.35 | 77.02 ± 15.99 | 75.75 ± 14.08 | 82.45 ± 7.64 | 2.455 | 0.141 | 0.209 | 0.172 | 0.990 |

| ***M. auratus* (AM)**  **‘dark morph’** | **AcTub^+^ fibers / cm (Posterior)** | | | | **ANOVA** | | **Tukey HSD *P* value** | | |
| --- | --- | --- | --- | --- | --- | --- | --- | --- | --- |
|  | **Dorsum** | **Medium** | **Ventrum** | **Whole trunk** | **F-value** | ***P* value** | **D vs. M** | **D vs. V** | **M vs. V** |
| **Fish 1** | 130.06 ± 21.28 | 115.51 ± 11.18 | 117.67 ± 7.26 | 121.64 ± 15.64 | 1.511 | 0.257 | 0.285 | 0.393 | 0.972 |
| **Fish 2** | 120.11 ± 11.16 | 116.06 ± 8.10 | 131.7515.80 | 122.23 ± 12.99 | 2.508 | 0.12 | 0.841 | 0.300 | 0.111 |
| **Fish 3** | 147.73 ± 26.50 | 103.74 ± 32.25 | 153.58 ± 21.91 | 135.02 ± 34.13 | 5.011 | <0.05 | 0.061 | 0.939 | <0.05 |
| **Fish 4** | 175.91 ± 21.81 | 131.33 ± 7.73 | 129.74 ± 17.67 | 145.66 ± 27.07 | 12.16 | <0.01 | <0.01 | <0.01 | 0.988 |
| **Fish 5** | 153.19 ± 20.21 | 140.75 ± 15.74 | 150.50 ± 26.10 | 148.15 ± 20.32 | 0.48 | 0.63 | 0.632 | 0.978 | 0.751 |
| **Average** | 145.40 ± 21.64 | 121.48 ± 14.56 | 136.65 ± 15.09 | 134.54 ± 12.52 | 2.241 | 0.131 | 0.116 | 0.713 | 0.382 |

| ***M. cyaneorhabdos***  **Female** | **AcTub^+^ fibers / cm (Posterior)** | | | | **ANOVA** | | **Tukey HSD *P* value** | | |
| --- | --- | --- | --- | --- | --- | --- | --- | --- | --- |
|  | **Dorsum** | **Medium** | **Ventrum** | **Whole trunk** | **F-value** | ***P* value** | **D vs. M** | **D vs. V** | **M vs. V** |
| **Fish 1** | 141.08 ± 30.37 | 103.36 ± 13.04 | 126.01 ± 26.55 | 123.64 ± 27.69 | 2.972 | 0.087 | 0.074 | 0.584 | 0.315 |
| **Fish 2** | 136.69 ± 13.22 | 130.58 ± 10.27 | 132.45 ± 10.00 | 133.24 ± 10.75 | 0.387 | 0.687 | 0.675 | 0.825 | 0.962 |
| **Fish 3** | 140.26 ± 24.01 | 113.34 ± 14.67 | 109.20 ± 45.68 | 120.94 ± 32.02 | 1.483 | 0.266 | 0.384 | 0.289 | 0.976 |
| **Fish 4** | 147.85 ± 21.77 | 85.92 ± 18.08 | 100.45 ± 17.64 | 111.41 ± 32.67 | 14.15 | <0.001 | <0.001 | <0.01 | 0.479 |
| **Fish 5** | 113.64 ± 34.41 | 108.01 ± 25.47 | 98.02 ± 10.88 | 106.5624.54 | 0.481 | 0.629 | 0.935 | 0.609 | 0.812 |
| **Average** | 135.90 ± 13.08 | 108.24 ± 16.18 | 113.23 ± 15.36 | 119.15 ±10.39 | 4.875 | <0.05 | <0.05 | 0.079 | 0.859 |

| ***M. cyaneorhabdos***  **Male** | **AcTub^+^ fibers / cm (Posterior)** | | | | **ANOVA** | | **Tukey HSD *P* value** | | |
| --- | --- | --- | --- | --- | --- | --- | --- | --- | --- |
|  | **Dorsum** | **Medium** | **Ventrum** | **Whole trunk** | **F-value** | ***P* value** | **D vs. M** | **D vs. V** | **M vs. V** |
| **Fish 1** | 130.84 ± 25.55 | 121.61 ± 17.72 | 107.41 ± 22.94 | 120.63 ± 23.28 | 1.476 | 0.264 | 0.781 | 0.237 | 0.593 |
| **Fish 2** | 136.53 ± 23.02 | 126.83 ± 18.17 | 107.39 ± 15.29 | 124.39 ± 22.01 | 3.126 | 0.078 | 0.694 | 0.067 | 0.288 |
| **Fish 3** | 139.89 ± 26.70 | 119.21 ± 37.40 | 78.71 ± 59.70 | 112.60 ± 48.10 | 2.56 | 0.119 | 0.738 | 0.107 | 0.338 |
| **Fish 4** | 138.12 ± 13.12 | 119.29 ± 3.99 | 97.60 ± 2.89 | 117.04 ± 21.32 | 12.75 | <0.001 | 0.102 | <0.001 | <0.05 |
| **Fish 5** | 133.48 ± 23.48 | 127.91 ± 9.89 | 106.73 ± 11.18 | 123.38 ± 19.57 | 3.744 | 0.052 | 0.849 | 0.050 | 0.152 |
| **Average** | 135.77 ± 3.63 | 122.97 ± 4.15 | 99.57 ± 12.38 | 119.61 ±4.84 | 27.52 | <0.001 | 0.058 | <0.001 | <0.01 |

**Supplementary Table S4. Statistical tests of axon density (across morph/sex)**

| **Species** | **position** | **AcTub+ fibers / cm (Anterior)** | | ***P* value**  **(two-tailed *t* test)** | **Fold change** |
| --- | --- | --- | --- | --- | --- |
|  |  | **dark morph / ‘dark morph’/ male** | **yellow morph / ‘yellow morph’/ female** |  |  |
| ***M. auratus* (WT)** | Dorsum | 143.51 ± 9.92 | 85.09 ± 18.84 | <0.001 | 1.69 |
|  | Medium | 124.38 ± 16.45 | 89.30 ± 9.35 | <0.001 | 1.40 |
|  | Ventrum | 153.32 ± 10.73 | 113.33 ± 16.52 | <0.01 | 1.35 |
|  | Whole trunk | 145.70 ± 7.16 | 95.93 ± 13.05 | <0.001 | 1.52 |
| ***M. auratus* (AM)** | Dorsum | 131.63 ± 7.79 | 97.61 ± 9.98 | <0.01 | 1.35 |
|  | Medium | 131.20 ± 14.83 | 89.30 ± 9.35 | <0.01 | 1.47 |
|  | Ventrum | 135.08 ± 8.39 | 93.80 ± 6.96 | <0.001 | 1.44 |
|  | Whole trunk | 132.60 ± 5.26 | 95.47 ± 4.58 | <0.001 | 1.40 |
| ***M. cyaneorhabdos*** | Dorsum | 124.38 ± 16.45 | 134.99 ± 15.87 | 0.330 | 0.92 |
|  | Medium | 116.30 ± 7.92 | 111.5413.66 | 0.524 | 1.04 |
|  | Ventrum | 104.91 ± 19.22 | 113.72 ± 16.90 | 0.464 | 0.92 |
|  | Whole trunk | 115.16 ± 5.58 | 120.07 ± 9.59 | 0.358 | 0.96 |

| **Species** | **position** | **AcTub+ fibers / cm (Posterior)** | | ***P* value**  **(two-tailed *t* test)** | **Fold change** |
| --- | --- | --- | --- | --- | --- |
|  |  | **dark morph / ‘dark morph’/ male** | **yellow morph / ‘yellow morph’/ female** |  |  |
| ***M. auratus* (WT)** | Dorsum | 154.93 ± 10.00 | 88.88 ± 17.65 | <0.001 | 1.74 |
|  | Medium | 136.27 ± 8.06 | 70.12 ± 8.61 | <0.001 | 1.94 |
|  | Ventrum | 143.50 ± 8.64 | 79.31 ± 13.96 | <0.001 | 1.81 |
|  | Whole trunk | 144.85 ± 3.55 | 79.25 ± 10.68 | <0.001 | 1.83 |
| ***M. auratus* (AM)** | Dorsum | 145.40 ± 21.64 | 94.58 ± 9.35 | <0.01 | 1.54 |
|  | Medium | 121.48 ± 14.56 | 77.02 ± 15.99 | <0.01 | 1.58 |
|  | Ventrum | 136.65 ± 15.09 | 75.75 ± 14.08­ | <0.001 | 1.80 |
|  | Whole trunk | 134.54 ± 12.52 | 82.45 ± 7.64 | <0.001 | 1.63 |
| ***M. cyaneorhabdos*** | Dorsum | 135.77 ± 3.63 | 135.90 ± 13.08 | 0.984 | 1.00 |
|  | Medium | 122.97 ± 4.15 | 108.24 ± 16.18 | 0.112 | 1.14 |
|  | Ventrum | 99.57 ± 12.38 | 113.23 ± 15.36 | 0.162 | 0.88 |
|  | Whole trunk | 119.61 ±4.84 | 119.15 ±10.39 | 0.933 | 1.00 |
